# Multifunctional mesoporous silica nanoparticles for biomedical applications

**DOI:** 10.1038/s41392-023-01654-7

**Published:** 2023-11-24

**Authors:** Bolong Xu, Shanshan Li, Rui Shi, Huiyu Liu

**Affiliations:** 1 Beijing Advanced Innovation Center for Soft Matter Science and Engineering, State Key Laboratory of Organic-Inorganic Composites, Bionanomaterials & Translational Engineering Laboratory, Beijing Key Laboratory of Bioprocess, Beijing Laboratory of Biomedical Materials, Beijing University of Chemical Technology, 100029 Beijing, China; 2 National Center for Orthopaedics, Beijing Research Institute of Traumatology and Orthopaedics, Beijing Jishuitan Hospital, 100035 Beijing, China

**Keywords:** Nanobiotechnology, Nanobiotechnology

## Abstract

Mesoporous silica nanoparticles (MSNs) are recognized as a prime example of nanotechnology applied in the biomedical field, due to their easily tunable structure and composition, diverse surface functionalization properties, and excellent biocompatibility. Over the past two decades, researchers have developed a wide variety of MSNs-based nanoplatforms through careful design and controlled preparation techniques, demonstrating their adaptability to various biomedical application scenarios. With the continuous breakthroughs of MSNs in the fields of biosensing, disease diagnosis and treatment, tissue engineering, etc., MSNs are gradually moving from basic research to clinical trials. In this review, we provide a detailed summary of MSNs in the biomedical field, beginning with a comprehensive overview of their development history. We then discuss the types of MSNs-based nanostructured architectures, as well as the classification of MSNs-based nanocomposites according to the elements existed in various inorganic functional components. Subsequently, we summarize the primary purposes of surface-functionalized modifications of MSNs. In the following, we discuss the biomedical applications of MSNs, and highlight the MSNs-based targeted therapeutic modalities currently developed. Given the importance of clinical translation, we also summarize the progress of MSNs in clinical trials. Finally, we take a perspective on the future direction and remaining challenges of MSNs in the biomedical field.

## Introduction

With the rapid development of nanotechnology, nanomaterials have shown great promise in the biomedical field due to their excellent physicochemical properties. A variety of nanoformulations have been widely explored and developed for cargo delivery,^1–5^ disease diagnosis,^6–9^ and therapeutic purposes.^10–13^ Compared to macroscale counterparts, nanoformulations always enjoy the unique merits, including higher bioavailability, reduced toxic effects and improved selectivity, in the living organism.^14^ Typically, nanoformulations include two major categories: organic and inorganic nanoformulations.^14,15^ Organic ones such as liposomes and polymers have been demonstrated to be a very effective and safe class of drug carriers, as evidenced by the fact that Doxil^l®^ is the first American Food and Drug Administration (FDA)-approved nanoliposomal drug formulation,^16^ and the recently reported development of two lipid nanoparticles (NPs)-based COVID-19 mRNA vaccines (BNT162b2 and mRNA-1273).^17^ For inorganic NPs-based formulations, although they are slightly inferior to organic NPs-based formulations in terms of biocompatibility and safety, they are superior to organic NPs-based formulations in terms of stability and drug delivery efficiency. Importantly, some unique properties possessed by inorganic NPs such as optical, ultrasonic, magnetic and catalytic properties have given rise to some novel NPs-based therapies, i.e., photothermal therapy (PTT),^18,19^ photodynamic therapy (PDT),^20,21^ sonodynamic therapy (SDT),^22,23^ chemodynamic therapy (CDT)^24,25^ and nanozyme-based catalytic therapy.^26,27^ Encouragingly, inorganic NPs are also gradually moving to the clinical stage, with about 25 inorganic nanomedicines approved for clinical use.^28^

**Fig. 1:**
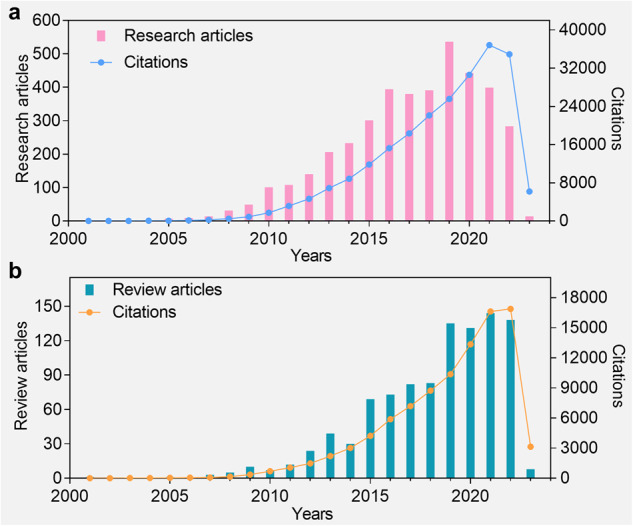
Summary of published literature on MSNs for biomedical applications, analyzed by Web of Science. **a** Research articles. **b** Review articles. Key words: “mesoporous silica” and “biomedical application”. Data collection for this statistic is up to March 2023

Among the different types of inorganic NPs-based formulations, mesoporous silica nanoparticles (MSNs) are of great interest to researchers worldwide due to their extreme flexibility in the manipulation of structure and properties. In recent years, the number of research papers on the applications of MSNs in the biomedical field has exceeded 300 per year (Fig. 1a). MSNs are characterized by a large range of tunable specific surface area and pore size, adjustable particle size and morphology, and easy surface functionalization. On the one hand, these features enable them to effectively load therapeutic drugs including small molecules, genes, peptides and proteins through electrostatic adsorption or chemical bonding, thus ultimately achieving targeted delivery and therapy.^29–31^ On the other hand, MSNs can act as substrate materials to load nanomaterials such as carbon dots,^32,33^ gold NPs^34,35^ and iron oxide NPs,^36,37^ resulting in inorganic nanocomposites with diverse properties to meet the requirements of various biomedical applications. In general, we can control the synthesis conditions to precisely modulate the topology with excellent internal and surface architecture of MSNs, and to obtain the desired performance.

An additional advantage of MSNs over other inorganic NPs is their relatively superior safety profile. A typical example is that the FDA has approved colloidal silica for use as a glidant in the production of tablets.^38^ Also, the widely used food additive E511 consists of amorphous silica NPs with a diameter of 100 nm.^38^ Importantly, there are numbers of clinical trials and clinical studies confirming the safety and efficacy of silica NPs when used in applications such as oral drug delivery, bioimaging and PTT. For example, in a clinical study involving 12 volunteers, ordered MSNs were able to significantly increase the bioavailability of fenofibrate by 54%, far better than the commercially available product Lipanthyl^®^.^39^ In another clinical trial involving 16 patients with prostate cancer, gold-silica nanoshells (GSNs) enabled tumor ablation by photothermal action, ultimately achieving effective tumor eradication in 94% (15/16) of patients.^40^ The physiological toxicity of MSNs is closely related to particle size, morphology, and structural composition. Currently, lots of silica-based nanoformulations have been developed, and their systematic safety evaluation is a matter of ongoing concern. However, there is no doubt that mesoporous silica-based NPs will always be more promising in the biomedical field than other inorganic NPs.

Although lots of reviews have already reported on the progress of MSNs in the biomedical field (Fig. 1b), the scope of this review provides a more comprehensive summary over the past decades from different aspects. Throughout this review, we provide an overview of the development history of MSNs in the biomedical field, introduce some key research advances, and summarize the various types of MSNs. We then summarize the nanocomposites composed of different functional inorganic components (i.e., metal compound NPs, noble metal NPs, upconversion NPs, and metal-free NPs) with MSNs. Our review focuses on the objectives of surface functionalization of MSNs, which include improving biocompatibility, enhancing targeting, and enabling precise drug delivery processes. We also highlight recent advances in MSNs-based biomedical applications, with particular emphasis on various types of targeted therapeutic strategies. Finally, we discuss the clinical translational status of MSNs and the current challenges they face in the biomedical field.

## Overview of MSNs

### Development history of MSNs

**Fig. 2:**
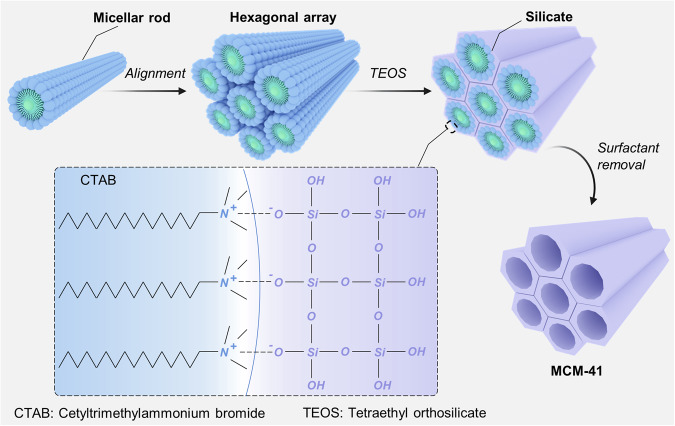
Schematic representation of MCM-41 synthesis. MCM-41 is prepared via surfactant-templating sol-gel method. CTAB surfactant is served as structure-directing agent, and TEOS is served as silica source. The mesoporous architectures of MCM-41 is determined by many factors including surfactant concentration, pH, and reaction temperature. The image elements were created using Autodesk 3ds Max

Silica possesses tetrahedral framework structure, which consists of a silicon atom and four oxygen atoms formed by covalent bonding. Mesoporous silica is a class of porous materials with the pore size distribution of 2–50 nm. In the preparation process of mesoporous silica, soluble silica precursors can be assembled into liquid-crystalline mesophases by adding the block copolymers or amphiphilic surfactants as the structure-directing agents.^29,41^ The silanes condensation followed by the structure-directing agents removal using solvent extraction or calcination methods results in the synthesis of amorphous mesoporous silica with different mesoporous phases (e.g., hexagonal, cubic, lamellar, and disordered structures).^42^ Research on mesoporous silica dates back to the 1960s, when some United States patents mentioned the preparation of mesoporous silica,^43,44^ but it was not until the 1990s that the study of mesoporous silica received increasing attention from researchers. In 1992, scientists from the Mobil Research and Development Corporation first synthesized a novel mesoporous material, Mobil Composition of Matter No. 41 (MCM-41), which is one member of the family of silicate-based mesoporous molecular sieves (M41S).^45^ MCM-41 exhibits an ordered hexagonal arrangement of uniform mesopores, and the channels of MCM-41 can be tailored in the range of 1.5–10 nm in size.^46^ In general, MCM-41 is prepared by using cetyltrimethylammonium bromide (CTAB) as the surfactant and Tetraethyl orthosilicate (TEOS) as the silica source (Fig. 2). Under strong alkaline condition, the surfactant initially forms micellar rods, and then stacks and arranges in hexagonal arrays. After adding the TEOS, the silicate in solution covers the hexagonal arrays to produce inorganic structure, in which the electrostatic interaction between negatively charged Si−O^−^ and positively charged −N^+^(CH_3_)_3_ results in the hydrolysis and condensation of silanes. Later, the calcination treatment leads to the removal of surfactant template to give the final product.^46^ At present, MCM-41 has become the most common nanomaterial used to build biomedical nanoplatforms.

**Fig. 3:**
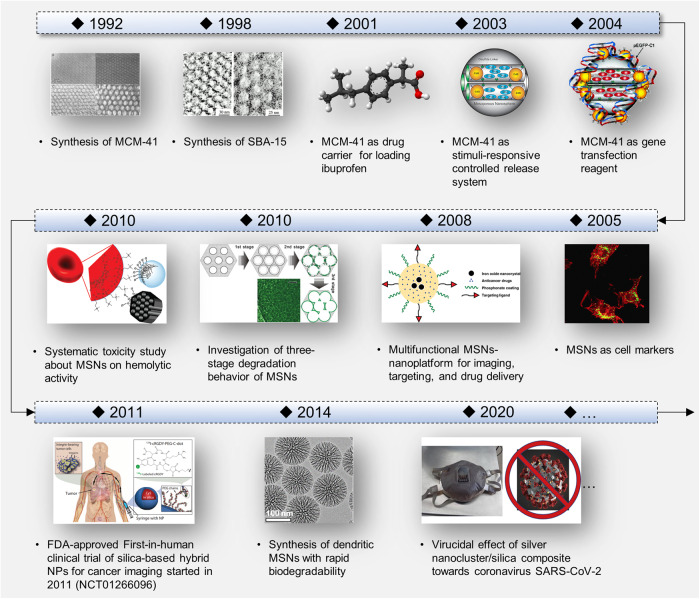
Timeline of the development history related to MSNs. Some key achievements are highlighted. Synthesis of MCM-41, image reprinted with permission.^46^ Copyright 1992, American Chemical Society. Synthesis of SBA-15, image reprinted with permission.^47^ Copyright 1998, The American Association for the Advancement of Science. MCM-41 as drug carrier for loading ibuprofen, image reprinted with permission.^48^ Copyright 2001, American Chemical Society. MCM-41 as stimuli-responsive controlled release system, image reprinted with permission.^51^ Copyright 2003, American Chemical Society. MCM-41 as gene transfection reagent, image reprinted with permission.^52^ Copyright 2004, American Chemical Society. MSNs as cell markers, image reprinted with permission.^53^ Copyright 2005, American Chemical Society. Multifunctional MSNs-nanoplatform for imaging, targeting, and drug delivery, image reprinted with permission.^54^ Copyright 2008, American Chemical Society. Investigation of three-stage degradation behavior of MSNs, image reprinted with permission.^55^ Copyright 2010, Elsevier. Systematic toxicity study about MSNs on hemolytic activity, image reprinted with permission.^56^ Copyright 2010, American Chemical Society. FDA-approved First-in-human clinical trial of silica-based hybrid NPs for cancer imaging started in 2011 (NCT01266096), image reprinted with permission.^58^ Copyright 2014, The American Association for the Advancement of Science. Synthesis of dendritic MSNs with rapid biodegradability, image reprinted with permission.^59^ Copyright 2014, American Chemical Society. Virucidal effect of silver nanocluster/silica composite toward coronavirus SARS-CoV-2, image reprinted with permission.^60^ Copyright 2020, Elsevier

Due to the important role of MSNs in the biomedical field, the timeline of some key achievements involving MSNs is presented in Fig. 3. Similar to MCM-41, Santa Barbara Amorphous-15 (SBA-15) is also demonstrated to be another very promising nanomaterial in the biomedical field, which is a highly stable mesoporous silica sieve reported by scientists from the University of California at Santa Barbara in 1998.^47^ Several years later, the first example of biomedical applications involving mesoporous silica for drug delivery is reported.^48^ MCM-41 is demonstrated to have the ability to load and deliver the anti-inflammatory drug, ibuprofen, with a weight percent ratio of 30%.^48^ In the same year, researchers from two different research groups both reported that the particle size of MCM-41 could be tuned to the nanoscale,^49,50^ and its morphology could also be precisely designed.^50^ These findings have greatly encouraged researchers to explore the potential biomedical applications of MSNs. In 2003, Lai et al. explored the feasibility of MCM-41-type MSNs in controlled-release delivery systems, in which they used cadmium sulfide (CdS) NPs as chemically removable caps to encapsulate drug molecules into the pore channels of MSNs.^51^ Subsequently, the disulfide bond-reducing molecules served as triggers to control the stimuli-responsive release of drug molecules including vancomycin and adenosine triphosphate.^51^ Meanwhile, the same research group also utilized polyamidoamines to modify the surface of MCM-41-type MSNs, and constructed a novel gene transfection system in 2004, which is the first study of the uptake behavior about MSNs into the eukaryotic cells.^52^ Afterwards, Lin et al. developed fluorescein-labelled hexagonal crystal-like MSNs with a size of 110 nm as cell marker.^53^ To fully exploit the physicochemical properties of MSNs and their potential for biomedical applications, in 2008, Liong et al. loaded superparamagnetic iron oxide into the internal pores of MSNs and subjected MSNs to phosphonate coating, targeting ligand modification and anti-cancer drug encapsulation, resulting in a multifunctional silica-based nanoplatform that can be used for imaging, targeting and drug delivery.^54^ Since the degradability of MSNs is critical to the development of nanoformulations with high safety, researchers continue to explore the degradation behavior and mechanisms of MSNs. In 2010, a three-state degradation process of MSNs was proposed, which presented a new understanding of the degradation kinetic mechanism of MSNs that differs significantly from that of traditional non-porous silica-based materials.^55^ Besides, Lin et al. systematically revealed the impact of MSNs with various particle size, pore structure and surface modification on hemolytic activity in 2010.^56^ This pioneering work provides guidance for understanding the toxic effects of MSNs in vivo. In 2011, Cornell dots, which are silica-based hybrid NPs with a size of 6–10 nm, become the first FDA-approved nanoformulation for a first-in-human clinical trial.^57^ The first-in-human clinical trial of ^124^I-labelled Cornell dots showed that they can be applied to diagnose and stage tumors including melanoma and malignant brain cancer.^58^ In addition, to obtain MSNs with rapid biodegradability, Zhao’s group developed a biphase stratification method for preparing monodispersed three-dimensional dendritic MSNs (3D-dendritic MSNs). The as-prepared 3D-dendritic MSNs can degrade completely in the simulated biological medium within 24 h.^59^ In recent years, with the outbreak and spread of coronavirus SARS-CoV-2, nanotechnology based on MSNs against coronavirus infection has also developed. In 2020, Balagna et al. performed preliminary antiviral test toward SARS-CoV-2 by using the silver nanocluster/silica nanocomposite deposited onto facial masks.^60^ In another work, a FDA-approved antiviral drug, niclosamide (NIC), was encapsulated into MSNs, followed by the coating with Tween 60. The MSNs-based nanocomposite was demonstrated to be a potential oral formulation for SARS-CoV-2.^61^

From these pioneering and outstanding works, we can witness that the research on MSNs covers all aspects of the biomedical field. With the further development of highly safe and efficient MSNs-based nanocomposites, as well as the systematic exploration of their in vivo biological action mechanisms, MSNs are gradually moving from basic research to clinical translation, contributing to the development of nanomedicine.

### Types of MSNs

In general, MSNs are often manufactured via surfactant-templating sol-gel method. Their structure and morphology are influenced by different factors, i.e., surfactants, silica sources, reaction catalysts, and other external reaction conditions such as pH and temperature.^62–64^ Of these, the surfactants as the structure-directing agents play a crucial role in determining the mesoporous architectures of MSNs, since they can induce the micellization of foam during the reaction process.^65^ Three main categories of structure-directing agents are frequently used in the synthesis of MSNs, including cationic surfactants (e.g., CTAB and cetyltrimethylammonium chloride (CTAC)), anionic surfactants (e.g., phosphoric acid, sodium dodecyl sulfate, and alkyl carboxylic acid), and non-ionic surfactants (Pluronic F123, F127, polyethylene oxide (PEO) and polypropylene oxide (PPO)).^66^ Due to the diversity of surfactants, various MSNs with unique configurations have been created and received phenomenal attention from researchers. At present, MSNs can be divided into M41S-series, SBA-series, Fudan University (FDU)-series, and Korea Institute of Technology (KIT)-series, etc, according to the mesoporous materials family. Table 1 summarizes some of the more well-studied types of MSNs. These different families of mesoporous silicas are detailed as follows.

**Table 1:** List of some representative types of MSNs

Types	Name	Syngony	Space group	Surfactant	Silica source	Synthesis condition	Ref.
MCM-series	MCM-41	2D hexagonal	*p6mm*	CTAB	TEOS	Basic condition	^45,46^
	MCM-48	3D cubic	*Ia3̄d*	CTAB	TEOS	Basic condition	^75^
	MCM-50	Lamellar	*p2*	CTAB	TEOS	Basic condition	^75^
SBA-series	SBA-1	3D cubic	*Pm3̄n*	C_16_TMA^+^(trimethylammonium)	TEOS	Acidic condition	^506^
	SBA-2	3D hexagonal	*P6*_*3*_*/mmc*	Gemini surfactant (C_n-s-1_)	TEOS	Acidic condition	^507^
	SBA-3	2D hexagonal	*p6m*	C_16_TMA^+^(trimethylammonium)	TEOS	Acidic condition	^75^
	SBA-11	3D cubic	*Pm3̄m*	Pluronic 123 (C_16_EO_10_)	TEOS	Acidic condition	^78^
	SBA-12	3D hexagonal	*P6*_*3*_*/mmc*	Pluronic 123 (C_18_EO_10_)	TEOS	Acidic condition	^78^
	SBA-15	2D hexagonal	*P6mm*	Pluronic 123 (EO_20_PO_70_EO_20_)	TEOS	Acidic condition	^47^
	SBA-16	3D cubic cages	*Im*3̄*m*	F127 (EO_106_PO_70_EO_106_)	TEOS	Acidic condition	^78^
FDU-series	FDU-1	3D cubic cages	*Im*3̄*m*	B50-6600 (EO_39_BO_47_EO_39_)	TEOS	Acidic condition	^91^
	FDU-2	3D cubic	*Fd3̄m*	C_m-2-3-1_	TEOS	Basic condition	^508^
	FDU-5	3D bicontinuous cubic	*Ia*3̄*d*	Pluronic 123 (EO_20_PO_70_EO_20_)	TEOS	Acidic condition	^509^
	FDU-12	3D cubic	*Fm*3̄*m*	F127 (EO_106_PO_70_EO_106_)	TEOS	Acidic condition	^94^
KIT-series	KIT-1	3D disordered mesostructure	/	CTAC	TEOS	Basic condition	^95^
	KIT-5	3D cage-like	*Fm*3̄*m*	F127 (EO_106_PO_70_EO_106_)	TEOS	Acidic condition	^96^
	KIT-6	3D bicontinuous cubic	*Ia*3̄*d*	Pluronic 123 (EO_20_PO_70_EO_20_)	TEOS	Acidic condition	^97^

#### M41S-series

As mentioned above, M41S-series mesoporous materials were firstly prepared by the Mobil Research and Development Corporation.^45,46^ The M41S series materials are typically characterized by a large amount of silanol groups (Si–OH) on both the internal pores and surface, the presence of which makes them easier to surface-functionalize for specific bioapplications. In addition, their mesophase arrangement, pore size, particle morphology and dimensions can be easily adjusted by changing the synthesis conditions. The most typical materials of the M41S series are MCM-41, MCM-48 and MCM-50,^67,68^ and those mesoporous materials can be synthesized by controlling the ratio of surfactants to silica source.^69^ As the well-investigated member of the nanostructured mesoporous materials, MCM-41 possesses two-dimensional (2D) hexagonal arrangements of unidirectional mesoporous pores, with *P6mm* space group symmetry.^45^ Different from MCM-41, MCM-48 shows the cubic arrangement containing Ia3d space-group symmetry, and it possesses a higher specific pore volume (up to 1.2 cm^3^ g^−1^), specific surface area (up to 1600 m^2^ g^–1^), and thermal stability.^70^ Accordingly, the three-dimensional (3D) pore structure and high porosity of MCM-48 makes it also advantageous in the field of drug delivery.^71–73^ The lamellar phase-MCM-50, which is separated by the surfactant layer to form a sandwich-like structure, has a *p2* space-group symmetry in the uncalcined form.^74,75^ When the surfactant is removed at high temperature, the lamellar structure of MCM-50 is unstable, which easily leads to the dense phases with little structural arrangement and porosity.^76^

#### SBA-series

SBA-series mesoporous materials were first reported by researchers from the University of California at Santa Barbara,^47^ which consists of a silica-based framework with highly ordered mesoporous structure, tunable pore size, high specific surface area, and thermal stability. There are many silica-based mesoporous materials in the SBA family, including SBA-1, SBA-2, SBA-3, SBA-6, SBA-7, SBA-8, SBA-11, SBA-12, SBA-14, SBA-15 and SBA-16.^47,63,77,78^ However, of these materials, only SBA-15 and SBA-16 are widely used in biomedical applications, while other types of MSNs such as SBA-1,^79–81^ SBA-2,^82,83^ SBA-3,^84,85^ SBA-11,^86,87^ SBA-12^88^ are mainly applied in the field of adsorption and catalysis. By using a triblock copolymer, Pluronic 123, as the structure-directing agent under acidic condition, SBA-15 with 2D hexagonal structure containing *P6mm* space-group symmetry can be synthesized.^47,89^ Compared with MCM-41, the thick pore walls (up to 9 nm) make SBA-15 more stable thermally and mechanically. Especially, the high specific surface area (~1000 m^2^g^–1^) and larger pore size (4–30 nm) of SBA-15 make it an excellent cargo carrier to load large molecule drugs in the biomedical field.^90^ Similarly, a cubic (*Im3̄m*) cage-structured SBA-16 can be obtained by using Pluronic F127 as the structure-directing agent, and the surface area and stability of SBA-16 are comparable to that of SBA-15.^78^

#### FDU-series

FDU-series mesoporous silica is mainly represented by FDU-1, FDU-2, FDU-5, and FDU-12, firstly reported by Zhao’s group from Fudan University.^91^ All of these possess 3D mesoporous architectures, well-ordered pore arrangements, amorphous pore wall structures and excellent thermal and mechanical stability.^62^ The first FDU-series MSN to be synthesized was FDU-1 in 2000, which has an *Im*3̄*m* space-group symmetry and exhibits a similar mesoporous structure to SBA-16.^91^ Unlike the SBA- and MCM-series MSNs, the FDU series have few bioapplications, and there are only a few reports of FDU-12 in the field of drug delivery.^92,93^ FDU-12 presents 3D cubic mesostructure with *Fm*3̄*m* space-group symmetry, and possesses a large cavity (10–12.3 nm), whose entrance sizes can be regulated in the range of ≈ 4–9 nm.^94^

#### KIT-series

The KIT-series was first reported by Ryoo’s group at Korea Advanced Institute of Science and Technology (KAIST),^95^ and the typical materials include KIT-1, KIT-5 and KIT-6. KIT-1 has disordered mesopores and amorphous pore walls, but its pore size is uniform and tunable, and it is more thermally stable than MCM-41.^95^ Meanwhile, KIT-5 and KIT-6 present well-ordered mesostructure with the space-group symmetry of *Fm3̄m* and *Ia3̄d*, respectively.^96,97^ Both the materials also have high specific surface areas, uniform pore size distribution and good stability, making them an excellent catalytic support. In addition, KIT-6 can be used to construct drug delivery systems for antimicrobial therapy, anti-tumor therapy and anti-*blastocystosis* therapy.^98–100^

#### Others

In addition to the aforementioned types of MSNs, other research groups have also reported the synthesis of MSNs with various mesoporous structures by changing the synthesis conditions, such as Institute of Bioengineering and Nanotechnology (IBN)-series,^101^ (AMS)-series,^102^ hexagonal mesoporous silica (HMS)-series,^103^ and Michigan State University (MSU)-series.^104^ These materials show similar structural characteristics to the SBA- and MCM-series MSNs, and thus have widespread applications in the fields of separation, adsorption, and catalysis.^105–109^ Since they have few studies in the biomedical field, we will not describe them much here.

## MSNs-based nanocomposites

**Fig. 4:**
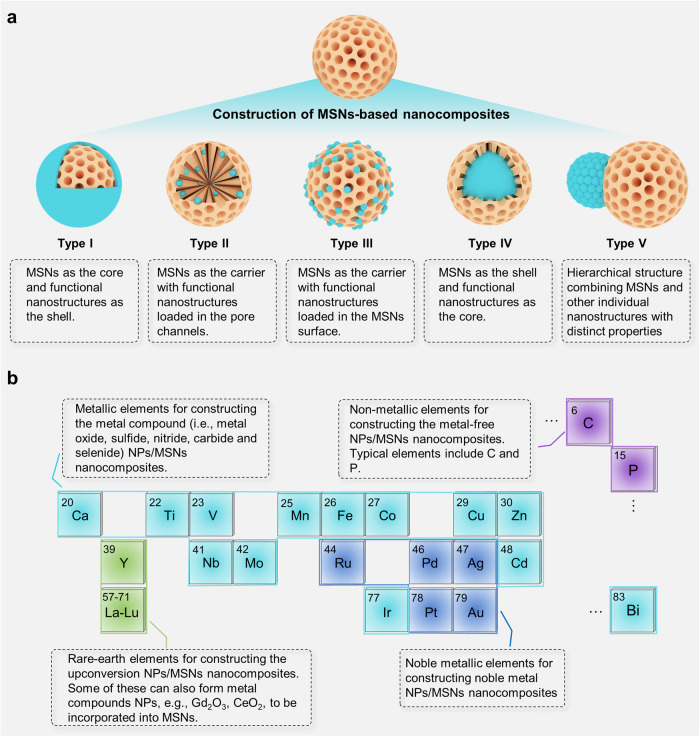
MSNs-based nanocomposites developed in the biomedical field. **a** Various nanostructured MSNs-based nanocomposites. Depending on the assembly process, the functional nanostructures can be introduced as the shell (Type I) or core (Type IV), can be loaded in the pore channels (Type II) or surface (Type III), and can form Janus-type hierarchical structure (Type V). **b** Typical elements used for constructing various types of MSNs-based nanocomposites. There are four main categories of nanocomposites based on the elemental type, including noble metal NPs/MSNs, metal compound NPs/MSNs, upconversion NPs/MSNs, and metal-free NPs/MSNs nanocomposites. The image elements were created using Autodesk 3ds Max

MSNs are a kind of relatively inert inorganic nanomaterials and are less frequently used as functional components for bioimaging or therapeutic purposes. However, as mentioned above, the features of MSNs including the high specific surface area, tunable pore size, controlled morphology and high mechanical/thermal stability, as well as good biosafety and biodegradability, make them excellent substrate materials for the construction of a wide range of nanocomposites.^110–114^ Through the host-guest assembly process, different kinds of inorganic functional components can be introduced to give the nanocomposites new physicochemical properties, such as magnetic-, light- and ultrasonic-response properties.^115–119^ Depending on the assembly strategy, specific nanostructured composites can be obtained, and there are five main types of MSNs-based nanocomposites (Fig. 4a): (1) Type I: Core-shell architectures,^120^ where the MSNs act as the inner core and the functional components as the outer shell. The functional nanoshells with specific sizes can be easily obtained by manipulating MSNs hard template. (2) Type II: Small-sized functional components loaded directly into the pores of MSNs. The common functional components such as carbon quantum dots and black phosphorus quantum dots are often encapsulated into MSNs in this form. In this architecture, MSNs enable slow and controlled release of small-sized functional components. (3) Type III: The functional component is loaded directly onto the surface of MSNs or on the periphery of the pore channel through covalent bonding or electrostatic adsorption. A distinct advantage of the Type III architecture is that it does not mask the active sites of the functional components, ensuring their catalytic stability. (4) Type IV: Core-shell architectures,^116,121,122^ but in which the MSNs act as the outer shell and the functional component acts as the inner core. Type IV architecture can avoid the aggregation of bare inorganic functional components, and afford the nanocomposites enhanced stability and decreased physiological toxicity. A common example is the upconversion NPs@MSNs nanocomposites, in which the MSNs are also often loaded with photosensitizers to synergize with the upconversion NPs for PDT.^122^ (5) Type V: Janus-type architectures. Janus-type nanocomposites have the biphasic geometries with distinct compositions or anisotropic structures, and the physicochemical properties between the individual components are largely unaffected,^123^ in contrast to the aforementioned Type I–IV nanocomposites.

According to the elements that act as the main components in inorganic functional nanomaterials, the currently existing types of MSNs-based nanocomposites for biomedical applications are summarized as follows.

### Noble metal NPs/MSNs nanocomposites

As an important branch in the field of inorganic nanomedicines, noble metal NPs have attracted widespread interest in the biomedical field from the very beginning. Noble metal NPs have made promising progress in areas including bioimaging,^124,125^ photothermal tumor ablation,^126–128^ PDT,^20,129,130^ radiotherapy sensitization,^131,132^ and recently developed nanozyme-based catalytic therapy,^133–135^ due to their tunable optical properties, excellent catalytic activity and good biocompatibility. Among those noble metal NPs, ruthenium (Ru),^136^ palladium (Pd),^137,138^ silver (Ag),^139,140^ platinum (Pt),^141,142^ and gold (Au)^143,144^ NPs have been reported to be loaded into MSNs to form various noble metal NPs/MSNs nanocomposites for biomedical applications (Fig. 4b). Of them, Au and Ag NPs are the most studied because of their relatively well-established synthetic routes, relatively high earthly reserves and good safety profiles.^145^

During the applications of noble metal NPs/MSNs nanocomposites, the corresponding noble metal elements can be properly selected and the aforementioned nanostructured architectures (Types I–V) or some new architectures can be rationally designed, according to the performance requirements and various therapeutic scenarios. For example, Au NPs are a promising photothermal agent due to their unique localized surface plasmon resonance (LSPR), but the bare Au NPs are not sufficiently stable during light irradiation, and suffer from poor colloidal stability under physiological conditions. To address this issue, a silica-protection strategy was developed, as reported by Duan et al., they coated mesoporous silica shell onto the surface of gold nanorods, and meanwhile additional gold nanoclusters were also incorporated on the surface of the mesoporous silica shell. The resulting core-shelled Au NP/MSNs nanocomposites (Type I architecture) achieved a photothermal conversion efficiency of 77.6%, significantly higher than that of bare gold nanorods.^146^ To overcome the cancer multidrug resistance (MDR) and enhance the penetration efficiency of nanomedicines, Kankala et al. designed a zinc metal species modified MSNs nanocarrier (Zn-MSNs), which could effectively disperse ultra-small Pt NPs, and the silica framework structure substantially promoted the loading efficiency of doxorubicin (DOX) (Type II architecture). During treatment, the ultra-small Pt NPs were able to penetrate deep into the tumor under the stimulation of acidic condition, and exhibited the peroxidase-like activity, decomposing hydrogen peroxide (H_2_O_2_) into toxic hydroxyl radicals (•OH) to kill tumor cells. Importantly, these consequences of the synergistic ablation of MDR cells by ultra-small Pt NPs were favorable only in the presence of the free radical generator, DOX.^147^ In addition, to design nanocomposites with high intelligence, e.g., in response to environmental stimuli or with self-propelling characteristic, the nanocomposites, asymmetric Janus-type nanostructures, have been created. Janus Ag/MSNs with SPR effect for pH-responsive drug delivery and SERS imaging,^139^ Janus Au/MSNs with the radiation absorption and SPR properties for CT and PA imaging of tumor,^148,149^ and Janus Pt/MSNs as an ultrafast self-propelled motion for smart drug delivery,^150^ have been reported successively.

### Metal compound NPs/MSNs nanocomposites

There is a wide variety of nanocomposites formed by metal compound NPs with MSNs, and the available noble metal-free elements include 13 elements, such as calcium (Ca),^151,152^ titanium (Ti),^153,154^ vanadium (V),^155,156^ manganese (Mn),^157,158^ iron (Fe),^159,160^ cobalt (Co),^161^ copper (Cu),^162,163^ zinc (Zn),^164,165^ Niobium (Nb),^166,167^ molybdenum (Mo),^168,169^ cadmium (Cd),^51,170^ iridium (Ir),^171^ and bismuth (Bi)^172,173^ (Fig. 4b). These metal elements can form metal oxide, sulfide, nitride, carbide and selenide, which exhibit different physicochemical properties and promising biomedical applications by combining with MSNs.^174–177^ Next we will take some typical examples as illustrations.

Among various metal oxide NPs/MSNs nanocomposites, the Fe_3_O_4_ NPs with magnetic targeting, magnetic hyperthermia, enzyme-like activity or Fenton reaction activity are the most studied functional nanostructures. Importantly, MSNs can also play a different role in the applications of these nanocomposites. For instance, MSNs that confine two different enzymes or enzyme mimics within the pore channel can be used as a nanoreactor for biomimetic cascade catalysis.^178,179^ Gao et al. encapsulated ultrasmall Au and Fe_3_O_4_ NPs into the pores of dendritic MSNs for the construction of a tumor microenvironment-responsive nanocatalytic reactor. In the spatially isolated Au-based and Fe_3_O_4_-based reaction chamber, Au NPs exhibited unique glucose oxidase-like activity, catalyzing the conversion of glucose to gluconic acid and H_2_O_2_, while the generated H_2_O_2_ was able to be utilized by the Fe_3_O_4_-based reaction chamber to boost the production of reactive oxygen species (ROS) by exhibiting peroxidase-like activity.^179^ In addition, MSNs are an ideal nanotherapeutic platform that can achieve synergistic treatment of Fe_3_O_4_-mediated catalytic therapy with other therapeutic modalities including PTT, PDT, and chemotherapy, by loading other therapeutic agents. As reported by Li et al., they utilized organo-mesoporous silica to load the ultrasmall Fe_3_O_4_ NPs and the photothermal agent Indocyanine Green (ICG). The photothermal effect caused by ICG could promote the Fenton reaction activity of Fe_3_O_4_ NPs, thus achieving amplified intracellular oxidative stress.^180^ Besides, Sun et al. reported the construction of core-shelled Fe_3_O_4_@MSNs that encapsulated DOX and 3-amino-1,2,4-triazole (AT). The MSNs-based therapeutic nanoplatform can realize enhanced anti-tumor efficacy by releasing chemotherapeutic drugs for the enhanced catalytic generation of ROS.^181^

For metal sulfide NPs/MSNs nanocomposites, the small-sized CdS NPs as a gatekeeper is the first example of MSNs-based therapeutic system for controlled drug delivery, reported in 2003.^51^ Other metal sulfides such as copper sulfide (CuS), molybdenum disulfide (MoS_2_) and bismuth sulfide (Bi_2_S_3_) exhibit high NIR absorption coefficients in the NIR region due to their plasmon resonance effect or electron-hole generation and relaxation mechanism,^182–186^ and thus those metal sulfides NPs can serve as potential photothermal agents. For instance, hollow MSNs functionalized with chitosan were synthesized, and then loaded with CuS nanodots. The CuS nanodots could act as a gatekeeper to seal the surface pores of MSNs, leading to the controllable release of DOX. The as-resulted CuS/MSNs-based therapeutic nanoplatform, with the photothermal conversion efficiency being 36.4%, dramatically extended the survival rate of tumor-bearing mice by the photothermal-enhanced synergistic therapy.^162^ Similarly, in another study, the MoS_2_ nanosheets with excellent photothermal conversion capability were also used as a capping agent to block MSNs to realize the drug release. Experimental results demonstrated that the MoS_2_/MSNs nanocomposite has the capability of pH-dependent and photothermal-triggered DOX release, thus achieving the targeted tumor killing by the combination of chemotherapy and PTT.^187^

In addition to the aforementioned metal sulfide NPs, the titanium nitride (TiN) NPs are also considered as a promising photothermal agent due to their high plasmonic absorption in the NIR region.^188^ It is worth noting that the silica coating may be beneficial to boost the photothermal conversion performance of TiN NPs.^153,189^ Gschwend et al. found that the plasmonic performance of silica-coated TiN NPs was much higher than that of bare TiN NPs, due to the reduced plasmonic coupling effects. The optimized nanocomposite showed a photothermal conversion efficiency of up to 58.5%, much higher than that of Au nanoshells that were used in the clinical trials and other commonly used inorganic photothermal agents.^153^ Besides, Chen’s group has reported the synthesis of a series of MSNs-coated transition metal carbide nanostructures, such as Nb_2_C and Ti_3_C_2_ with different surface modifications.^166,167,175,190,191^ These nanocomposites were demonstrated to show excellent photothermal properties, and realized the efficient combination of multiple therapeutic modalities through MSNs-mediated targeted drug delivery, thus providing some new solutions for the disease diagnosis and treatment.

### Upconversion NPs/MSNs nanocomposites

Upconversion NPs are a class of lanthanide ions-doped inorganic nanomaterials that can absorb low energy light, and then emit high energy light (visible or ultraviolet light) through the anti-Stokes effect.^192–194^ The rare earth elements are the main components used to prepare upconversion NPs (Fig. 4b).^195^ The unique optical property of upconversion NPs, i.e., converting NIR light with high biological tissue penetration into the visible or ultraviolet light, can be used for the photosensitiser excitation in PDT or serve as the contrast agents in bioimaging.^196–201^ In general, the upconversion NPs consist of three main parts, namely the activator, sensitizer and host.^202^ The common activators include trivalent ions such as Pr^3+^, Nd^3+^, Er^3+^, and Tm^3+^. These ions have abundant ladder-like energy levels and their spectra are less affected by the host.^203^ Yb^3+^ is the most commonly used and effective sensitizer, with a large absorption cross-section at about 980 nm and a good match with the absorption spectra of the activator,^204^ which can significantly promote the energy transfer efficiency between the activator and Yb^3+^ sensitizer.^205–208^

The upconversion NPs are characterized by large anti-Stokes shifts, narrow emission bandwidths, and minimal spectral overlap with tissue autofluorescence.^209,210^ To modify the surface hydrophilicity of upconversion NPs to provide stable aqueous colloidal dispersions, and obtain the ability to conjugate biomolecules and other ligands on the upconversion NPs, a common approach for researchers is to coat the upconversion NPs surface with a layer of MSNs.^211,212^ Such constructed upconversion NPs/MSNs nanocomposites-based platform offers a wide range of applications of the upconversion NPs in the biomedical field.^212,213^ In bioimaging applications, the coating of MSNs is able to not only reduce particle aggregation and enhance particle stability, but also modulate the contrast properties of upconversion NPs.^214,215^ In Gd^3+^-doped core-shelled upconversion NPs/MSNs nanocomposite, the outer MSNs with flexible and adjustable shell layer thickness and porosity can regulate the coordination number, residence time and rotational correlation time of water molecules, thus affecting the relaxation mechanism and magnetic resonance sensitivity of upconversion NPs.^215^ In the construction of therapeutic systems, the drug delivery capability of MSNs can be used to achieve NIR-mediated PDT, chemotherapy or multimodal synergistic therapy. For example, NaYF_4_:Yb/Er, an upconversion nanomaterial that produces both green and red emission under NIR light excitation, enables dual photosensitisers activation at a single NIR light excitation by encapsulating both merocyanine 540 (MC540) and zinc (II) phthalocyanine (ZnPc) photosensitisers into the surface layer of MSNs, thereby achieving enhanced ^1^O_2_ production.^216^ In another study, to address the clinical aspects of therapeutic agents in thrombolytic therapy, a NIR-mediated upconversion NPs/MSNs therapeutic drug delivery platform was constructed. The upconversion NPs emit UV/blue light upon excitation by 808 nm NIR light, which could fuel azobenzene to propel the release of urokinase (UK), as well as induce the responsive release of NO, resulting in effective synergistic thrombolytic and anticoagulation therapy.^217^

### Metal-free NPs/MSNs nanocomposites

Among the non-metallic elements, two elements, carbon and phosphorus, can be used to construct carbon-based nanomaterials (e.g., graphene,^218–220^ carbon dots,^221–223^ carbon nanotubes^224–226^ and amorphous carbon^227–229^) and black phosphorus (BP),^230–232^ respectively, resulting in metal-free NPs/MSNs nanocomposites (Fig. 4b). Compared to metal- or metal compounds-based nanomaterials, metal-free NPs can largely reduce potential metal ion-induced toxic effects.^233,234^ The delivery of these functional metal-free NPs via MSNs often also allows for desired therapeutic effects comparable to or even higher than those of metal- or metal compound-based nanomaterials.

For carbon-based nanomaterials, they can serve as photothermal agents for photothermal antibacterial and photothermal tumor ablation due to their broad-spectrum light-absorption properties.^235–238^ However, the disadvantages such as poor solubility and negative material-biological interface interactions limit their further applications.^239,240^ To address this issue, MSNs-coated carbon-based nanomaterials have been created. Such-constructed nanocomposites not only improve the surface interface properties of carbon-based materials, but also combine the advantages of two different drug carriers, including the enhanced water solubility and dispersibility, easier surface functionalization properties and higher drug loading and delivery performance.^219,241^ Another problematic aspect of carbon-based materials is that their in vivo biodegradability is difficult to manipulate, which may bring some potential long-term physiological toxicity issues.^242^ In tumor therapy, although NPs smaller than 5.5 nm can usually be cleared from the body by the renal metabolic system, particle sizes too small to be enriched to tumor sites by the enhanced permeability and retention (EPR) effect when performing administration intravenously.^243–245^ For this reason, researchers can use the MSNs with excellent biodegradable properties to address these issues of carbon-based nanomaterials.^229^ A recent study showed that by coating a carbon layer on the surface of dendritic degradable MSNs, accelerated degradation of MSNs and collapse of the surface carbon layer could be observed with NIR irradiation. This carbon-silica nanocomposite was rapidly cleared from the body after completion of a synergistic PTT and PDT.^229^

BP is a 2D semiconductor nanomaterial, commonly found as quantum dots and nanosheets,^246–248^ whose excellent NIR photocatalytic activity and broad-spectrum light-absorption give it photodynamic effect and photothermal conversion capability, respectively.^249,250^ Since BP nanosheets have a large number of lone-pair electrons in their own structure, which are difficult to stabilize in the presence of water and oxygen, BP nanosheets exhibit rapid biodegradability under physiological conditions and can be degraded to less toxic phosphate species.^232,251^ In addition, BP nanosheets tend to exhibit a hydrophobic surface, which is poorly dispersed and not easily functionalized under physiological conditions.^252,253^ However, the formation of BP/MSNs nanocomposites, on the one hand, reduces the rate of degradation of BP and prevents its premature clearance through renal excretion, on the other hand, have a more stable drug loading capacity and an effective modification potential.^230^ Besides, MSNs also possess the ability to regulate the physicochemical properties of BP. For example, the MSNs encapsulated on the surface of BP nanosheets could improve the photoluminescence lifetime of BP to some extent by affecting the local microenvironment, resulting in an extended photoluminescence lifetime, significantly higher than that of pure BP NPs.^254^

## SURFACE functionalization

MSNs have very competitive applications in various areas of biomedicine, to a large extent due to their hydrophilic surface containing a large proportion of Si–OH groups, which makes them susceptible to various functionalization modifications on the external or internal porous surface.^255,256^ Surface functionalization to adjust the physicochemical properties of MSNs is expected to overcome some of the shortcomings of MSNs, or to make them smarter in their applications, adapting them to changes in response to different application scenarios and external stimuli.^257,258^ For example, while MSNs can improve the drug stability and delivery efficiency, it is still of interest to ensure that the drug is protected from enzymatic degradation and to avoid premature release.^2,259^ In this case, attempts have been made to address this issue by coating the MSNs surface with polymers and lipid bilayers, or by introducing environmentally responsive factors.^260–264^ Surface-functionalized modifications of MSNs not only offer unique advantages in terms of improved biosafety, long circulation and targeting ability, but can also be used to construct intelligent stimuli-responsive drug delivery systems.^265–269^ Once they reach the lesion sites, the spatial and temporal controlled release of drugs in response to stimuli such as pH,^270,271^ temperature,^272,273^ light^274–276^ and ultrasound^277,278^ can be achieved. We next will describe the purpose of surface functionalization of MSNs.

### Improving the biosafety of MSNs

**Fig. 5:**
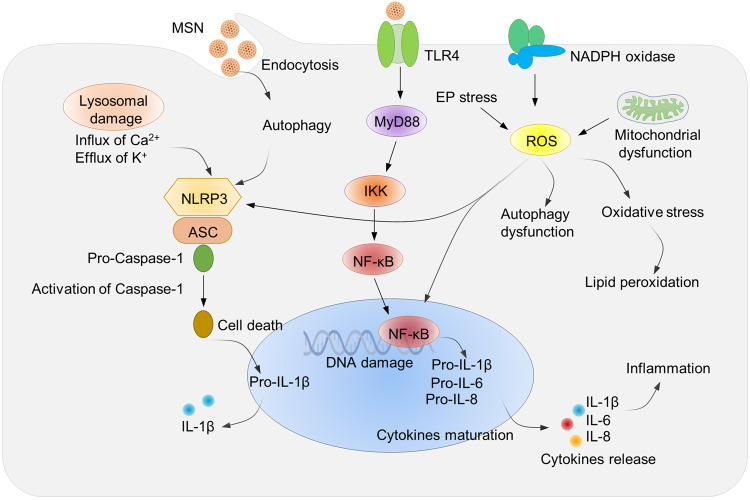
The main mechanism of toxicity mediated by silica NPs. The prominent mechanisms of toxicity for silica NPs include autophagy, oxidative stress, and pro-inflammatory response

Although the biosafety of MSNs has been significantly improved compared to other inorganic NPs, their toxicity mechanism study cannot be ignored. In general, the toxicity of silica NPs is highly related to their size, morphology, surface charge, crystallinity and dose.^56,279–282^ For example, it was shown that MSNs with positive surface charge were able to induce stronger ROS-mediated toxic effects than MSNs with other charges.^283–285^ In addition, Napierska et al. showed that MSNs with particle size larger than 100 nm exhibited low cytotoxicity, while those smaller than 50 nm could induce obvious cell death.^286^ The toxicity of MSNs to normal cells can be attributed to two aspects: (1) Elevates intracellular oxidative stress by inducing the production of toxic ROS and decreases the expression level of glutathione, which has a role in regulating redox homeostasis, leading to lipid peroxidation and subsequent cell death.^287,288^ (2) The unbonded Si–OH groups on the surface of MSNs interact electrostatically with phospholipids on the cell membrane surface, leading to the damage of cell membrane.^284,289^ In addition, bare MSNs are less stable under the physiological ionic strength, are prone to aggregation,^290^ and are rapidly removed from the circulation by nonspecific binding and uptake by the immune system. Currently, the most prominent mechanisms of toxicity for silica NPs include autophagy, oxidative stress, and pro-inflammatory response (Fig. 5).^291^ For instance, studies have pointed out that environmental exposure to silica NPs can cause the ROS-mediated autophagy dysfunction and cell apoptosis through MAPK/Bcl-2 and PI3K/Akt/mTOR signaling pathways.^292^ The NLRP3- inflammasome-activation pathway underlies the asbestosis and silicosis.^293^ The NF-κB signaling pathway, activated by silica NPs in many types of cells, leads to the upregulation of inflammatory gene and autophagy-related cell death.^291,294^

**Fig. 6:**
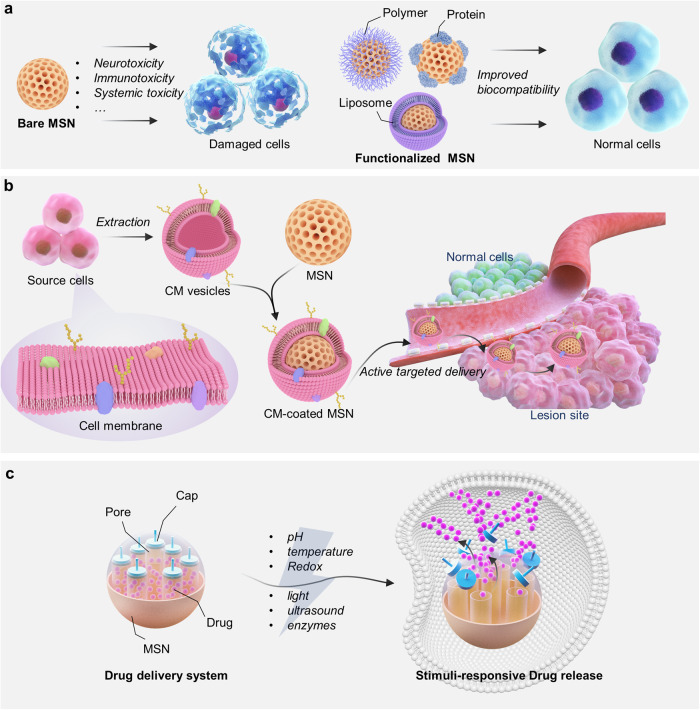
Surface-functionalized modifications of MSNs for different application purposes. **a** Coating MSNs with polymer, protein, or liposome to improve the biosafety of MSNs, and avoid the potential toxicity effect induced by bare MSNs. **b** Surface modification of MSNs through cell membrane-based biomimetic strategy to improve the target ability of MSNs. **c** Surface modification of MSNs using cap agents to impart the stimulus-responsive properties to MSNs. The image elements were created using Autodesk 3ds Max

Considering these factors, substantial efforts have been carried out to go for specific surface functionalization modifications of MSNs to decrease their toxicity such as neurotoxicity, immunotoxicity, and systemic toxicity (Fig. 6a). A common approach is to integrate the biocompatible polymers with MSNs. Polymers including polyethylene glycol (PEG), polyethylenimine (PEI) and chitosan have been demonstrated to be excellent surface coating agents for MSNs, which can significantly improve the in vivo circulation time, reduce cytotoxicity and hemolytic effect of MSNs.^295–298^ In terms of reducing protein adsorption and improving the colloidal stability of the particles, PEG is currently considered as a very excellent polymer.^298–300^ Hao et al. systematically investigated the hemolytic activity and protein adsorption behavior of bare MSNs, MSNs modified with chitosan (MSNs-CS) and MSNs co-modified with chitosan and PEG (MSNs-CS-PEG).^301^ Results showed that the hemolysis percentage and protein adsorption of MSNs-CS-PEG were 2% and 3.2%, respectively, which were remarkably lower than those of bare MSNs (30.9% and 14.5%, respectively), and they also exhibited lower toxicity toward MCF-7 cells.^301^ This was similarly demonstrated in a study by He et al.^302^ In addition to polymers, liposomes have the advantages of high biocompatibility, low immunogenicity and long circulation, thereby they can also be used to improve the biosafety of MSNs.^303–305^ MSNs can be encapsulated by lipid bilayers or multilayers, and these liposomes act as protective shells to reduce toxicity by masking the reactive groups on the surface of MSNs. Compared to bare MSNs, liposome-encapsulated MSNs exhibit superior particle dispersion and lower non-specific binding.^306,307^ Moreover, they lead to the higher bioavailability and the in vivo half-life can be prolonged more than 10-fold, reducing the distribution of MSNs in reticuloendothelial system (RES)-related organs.^306^ Protein coating is also a way to improve the biocompatibility and particle stability of MSNs.^308,309^ Negatively charged bull serum albumin (BSA), which is highly physiologically stable during blood circulation, can tightly bind to the amino groups on the MSNs surface, preventing the premature release of drug loaded on the MSNs.^309^ Besides, given that the positively charged MSNs show significant toxicity effect, some researchers have devoted to regulating the surface properties of MSNs by direct group modification. The surface potential of MSNs can be regulated by various functionalization via carboxyl (–COOH), phenyl (–Ph), and methyl phosphonate (–PO_3_^−^) groups with negative and neutral zeta potentials.^310,311^

### Increasing the targeting ability of MSNs to the lesion site

The pathway of administration of MSN during treatment includes oral, inhalation, intravenous, intramuscular and intraperitoneal injections. Regardless of the administration method, it is expected that the MSNs will be maximally enriched at the lesion site. However, in those delivery systems, MSNs-based formulations tend to distribute throughout the body.^312,313^ Some studies have shown that MSNs accumulate mainly in the liver and spleen,^312,314,315^ and that the high concentration of MSNs at these normal organ tissues not only severely reduces the therapeutic efficacy of MSNs-based formulations, but also induces some potentially toxic effects on the RES. Therefore, enhancing the active targeting ability of MSNs has significant implications for facilitating preclinical studies of MSNs-based therapeutic system.

Targeted delivery strategies are generally divided into two categories: passive targeting and active targeting. Passive targeting relies on the pathological characteristics of the disease microenvironment and the nature of the drug delivery system itself, which allows the drug to effectively accumulate at the lesion site of disease. The most well-known passive targeting is the EPR effect of tumor, which refers to the phenomenon that some particles with specific size (20–200 nm) penetrate more easily into tumor tissue and remain there for a long time when compared to normal tissue.^316,317^ Therefore, controlling the size of MSNs to a suitable range will facilitate their enrichment in the tumor sites. However, it has also been pointed out that the passive targeting strategy relying on the EPR effect can achieve only 0.7% of the injected NPs enrichment in solid tumors,^318^ which will not be beneficial for the clinical translation of nanotechnology. In contrast, active targeting relies on the active recognition between the molecules on the NPs surface and the specific molecules or proteins in the disease microenvironment, which is also referred to as ligand-receptor specific binding.^319^ The active targeting shows a higher drug delivery efficiency compared to passive targeting.^320^ Targeted ligands, such as proteins, antibodies, peptides, nucleic acids and chemical small molecules, can be modified on the surface of MSNs, and the modified MSNs are capable of selectively aggregating at the lesion site based on the strong affinity between the targeted ligands and the specific receptors overexpressed in the disease microenvironment.^321–324^ For example, compared to mature vascular endothelial cells, tumor neovascular endothelial cells are highly expressed in a variety of proteins, including integrins, transmembrane glycoproteins and aminopeptidase N. Molecules that recognize these highly expressed proteins can be used for neovascular-based targeted drug delivery. A typical example is that arginylglycylaspartic acid (RGD)-modified MSNs can selectively bind integrin α_ν_β_3_ receptor and thus be applied to target tumor neovascularization.^325^

In addition to targeted ligand, cell membrane-based biomimetic strategy is also a current and very feasible approach to improve the targeting ability of MSNs (Fig. 6b). The cell membrane biomimetic technique dates back to 2011, when Zhang’s group used intact red blood cell membranes to encapsulate NPs via a top-down strategy. Compared with unencapsulated NPs, NPs encapsulated by red blood cell membranes have a longer half-life in mice and remain in circulation for up to 72 h due to the immune escape function possessed by the cell membrane.^326^ The preparation process is generally divided into three steps: membrane extraction, preparation of inner core NPs, and fusion of membrane and NPs. The cell membrane-coated NPs combine the advantages of outer cells and inner core NPs, and achieve long-term in vivo circulation and targeted delivery while greatly improving biocompatibility.^327^ The therapeutic potential of various types of cell membranes-engineered MSNs-based drug delivery systems has been demonstrated in a variety of disease models. For example, platelet membrane-coated MSNs were used for early atherosclerosis therapy by mimicking the immune escape ability of platelet.^328^ Cancer cell membrane-coated MSNs were specifically enriched in tumor sites by homologous targeting properties, and activated the ferroptosis-related immunogenic cell death on gastric cancer.^329^ Bacterial outer membrane-coated MSNs are used for targeted delivery of the antibiotic rifampicin to achieve in vivo resistance to gram-negative bacterial infections.^330^ Despite the limitations of current research related to cell membrane biomimetic strategy, these examples invariably demonstrate the effectiveness of cell membrane-based active targeting strategy in improving the targeting ability and therapeutic efficacy of MSNs.

### Controlling the drug delivery of MSNs

MSNs are gradually being developed as promising drug delivery carriers due to their excellent biocompatibility, biodegradability, and highly ordered pore structure. Due to the great flexibility in tuning the surface properties of MSNs, specific surface functionalization modification of MSNs can achieve precise modulation of drug delivery behavior, thus to meet the application requirements. Currently, the most researched is the construction of stimuli-responsive MSNs-based drug delivery system (Fig. 6c), i.e., when MSNs reach the lesion site, the release of drugs is controllably stimulated by specific internal or external factors, thus achieving the improved drug utilization and avoiding drug leakage at non-lesion sites.^259,272,331^ The most core step in this process is connecting some caps with stimulus-responsive properties onto the MSNs surface, which can be called gatekeepers,^119,158^ nanovalves,^332,333^ or others. Once the therapeutic drug is filled into the mesopore of MSNs, the pore entrances are blocked by the caps to prevent the drug from diffusing out. Under normal physiological conditions, the caps always keep the pores in a closed state, but when reaching to the lesion, the caps are separated under the stimulation of certain factors, which in turn induces targeted on-demand release of drugs. These caps can be polymers,^334^ metal/metal compound NPs,^170,335^ biomolecules,^336^ etc. While exogenous stimuli such as light, ultrasound, magnetic field and electric field, endogenous stimuli such as temperature, pH, redox agents, and enzymes, can activate the caps. Several examples about the surface modification of MSNs and the corresponding stimuli-responsive strategies are briefly described here.

The pH in normal tissues under physiological conditions is generally neutral, while in solid tumors, the tumor microenvironment is generally slightly acidic due to the exuberant metabolism of tumor cells that leads to the production of large amounts of lactic acid.^337,338^ Therefore, the construction of pH-responsive MSNs nanocarriers can achieve targeted drug release and tumor therapy. Wagner et al. functionalized the external surface of MSNs by carboxylic groups, followed by attaching a stimuli-responsive capping system, that consists of a pH-responsive acetal linker and a biotin–avidin gatekeeper.^339^ After uptaken by lysosome in tumor environment, the biotin–avidin caps were separated out and the immune-stimulant R848 (resiquimod) could be released, thus achieving enhanced targeted delivery of immune modulator to antigen-presenting cells.^339^ Besides, in the construction of a light-responsive drug delivery system, Zhao et al. grafted the light-responsive azobenzene group on the surface of biodegradable MSNs, and β-cyclodextrin-modified polymer as the cap agent. In vitro experiments verified that the visible light triggered the isomerization of azobenzene, followed by the dissociation of CD-PMPC from MSNs surface and subsequent drug release. The excellent anti-inflammatory effect of MSNs-based platform demonstrated their potential in the treatment of osteoarthritis.^274^ Regarding enzyme-responsive drug delivery system, some researchers reported the synthesis of MSNs attached with chitosan gatekeeper via azo bonds. The azo bonds could be cleaved by the colon-specific enzyme, leading to the separation of chitosan gatekeeper and the release of DOX.^334^ In all, these functionalized MSNs-based drug delivery systems that specifically respond multiple stimulus signals offer a precisely localized and targeted way for disease treatments.

## Biomedical applications

MSNs are chemically and biologically inert nanomaterials compared to other inorganic nanomaterials. Only a few studies have been reported on the biomedical applications based on the inherent activities of MSNs. For example, it has been pointed out that the MSNs containing rich pore structure possess ultrasound-induced cavitation effect, and they exhibit sonodynamic activity by moderately modulating the surface wettability for thrombolysis therapy.^340^ In addition, Si ions have been demonstrated to show some natural bioactivity in tissue engineering, and the Si ions released from the degradation of MSNs can activate the expression of bone-related genes or proteins, stimulate the cartilage differentiation, and thus play an important role in the formation process of bone and cartilage.^341^ However, given their relatively low outcome efficiency, the current research on MSNs in the biomedical field is more focused on acting as matrix to form nanocomposites, or as nanocarriers to deliver various cargos. In this part, the various types of MSNs-based nanomaterials in biosensing, bioimaging, targeted disease therapy and tissue engineering will be summarized (Table 2).

**Table 2:** List of some representative examples of biomedical applications of MSNs

Material	Composition	Application	Method/Strategy	Ref.
cDNA-gated MSNs	cDNA is grafted to the aminated MSNs with an average size of 50 nm	Detecting CEA	cDNA-gated enzymatic reaction induces fluorescence quenching	^343^
MSN@CUR@ZnO@pAbs	CUR-loaded MSN is capped with ZnO, and modified with pAbs	Detecting Salmonella	Colourimetric and fluorescence detection using CUR as signal reporter	^344^
UCNPs@mSiO_2_	UCNPs are encapsulated by MSNs into core-shell structure with a shell thickness of 15 nm	Detecting CRP	Fluorescence detection using luminescent UCNPs as signal reporter	^345^
MSNs@4-ATP-Apts	4-ATP is encapsulated into the pore of aptamers- grafted MSNs	Detecting Staphylococcus aureus	SERS technology for measuring the signal intensity of aptamers-gated released 4-ATP	^346^
Probe-gated MSNs	DNA probe is immobilized onto the surface of aminated MSN filled with fluorescein	Detecting SARS-CoV-2 genome	Fluorescence detection of DNA probe-gated released fluorescein molecules	^347^
MB@MPSF	MB-encapsulated MPSF is capped with aptamer probes	Detecting SARS-CoV-2-RBD	Electrochemical signal of aptamers-gated released MB	^349^
FMH NPs-HE	FITC-doped rattle-type silica colloidal particles are loaded with HE	Detecting Superoxide anion	Ratiometric fluorescence based on FITC (Ex/Em: 470/518 nm) and superoxide anion HE (Ex/Em: 518/570 nm)	^510^
Cu-MSN	Copper-modified MSNs with average sizes of 30–300 nm	Detecting GSH and H_2_O_2_	UV-Vis spectroscopy in the presence of chromogenic substrate	^511^
CD@DFNS@SH	SH- immobilized DFNS (particle size: ∼50 nm, surface area: 1080 m^2^/g) is loaded with CDs (particle size: 5–7 nm)	Detecting mercury ion in living Artemia Salina	Fluorescence detection using red emissive CDs as signal reporter	^512^
VMSN	Vanadium oxide NPs (particle size: 3–5 nm) are dispersed throughout the MSNs (particle size: ∼45 ± 10 nm)	Detecting dopamine	Colorimetric detection based on the oxidase-like activity of VMSN	^513^
^64^Cu-sulfur-SNP	Thiol group-functionalized 130 nm SNP labeled with ^64^Cu	PET imaging of lymph node	Thiol group enhances the binding ability of ^64^Cu to silica matrix, and improves the PET imaging stability	^355^
^18^F-MSNs	Aminated MSNs (particle size: 30–130 nm) functionalized with *N*-succinimidyl 4-[^18^F]fluorobenzoate	PET imaging for evaluating in vivo biodistribution	MSNs-protected ^18^F positron emission isotope for PET imaging	^356^
Au@mSiO_2_-PFH-PDA	Au core/hollow MSNs (shell thickness: 22.9 ± 2.2 nm) coated with PDA and filled with PFH	PA/US/CT/thermal imaging of tumor	MSNs-protected Au core for CT imaging, PFH for US imaging, and PDA for PA/thermal imaging	^358^
Cy754-MSNs	Cy754 dye-doped MSNs with an average size of 35 nm	FL and PA imaging of SLNs	Dye-loaded MSNs simultaneously produce moderate NIR emission and PA signals	^359^
MTX-ICG-MSNs	ICG-conjugated MSNs (particle size: 25–50 nm) loaded with MTX	PA imaging for monitoring drug release	PA_700 nm_/PA_810 nm_ ratio for quantifying the MTX release from MSNs	^360^
FMSNs-DOX	DOX-loaded FMSNs with particle size and contact angle being 217 ± 58 nm and 129 ± 3°, respectively	US imaging of tumor	Superhydrophobic PFDTS on the MSNs surface stabilize the interfacial nanobubbles for the contrast enhancement of US imaging	^361^
D-MON-RGD	Gd-DTPA-loadeded D-MON modified by RGD	MRI imaging of GL261 tumor	The deformability of MSNs increases the blood circulation, and thus improve the MRI contrast performance	^514^
CsPbBr_3_@SiO_2_	CsPbBr_3_ perovskite nanocrystals coated with SiO_2_ (shell thickness tunesd from 9 to 51 nm)	FL imaging of HeLa cells	MSNs enhance the stability of CsPbBr_3_ without decreasing the PL emission	^515^
MSNs-C	Cell membrane-coated MSNs (particle size: ∼100 nm) loaded with DOX	Treatment of breast tumor	Functionalized cell membrane and chemotherapeutic drug DOX for targeted tumor immunotherapy and chemotherapy, respectively	^367^
DOX@Au@MMSN-Ald	Ald-modified Au@MMSN loaded with DOX (hydrodynamic diameter: 174.1 nm)	CT/MRI imaging, and treatment of osteosarcoma	Au and Mn for CT and MRI imaging, respectively; Ald, DOX, and Mn for preventing osteolysis, chemotherapy, and chemodynamic therapy, respectively	^382^
AuNR@MS	Mesoporous silica-coated Au nanorod modified with PEG (hydrodynamic diameter: 86.0 nm)	CT imaging and tumor therapy	Au nanorod as X-ray contrast agent for CT imaging, and as radiosensitizer for radiotherapy with cobalt 60 source	^383^
DLMSN/CuS/RBCc/RBCm	RBCm-coated, CuS and RBCc co-loaded DLMSN (particle size: 102 ± 8 nm)	Treatment of mammary carcinoma	Combination of PTT and radiotherapy based on CuS NPs; RBCc as an oxygen reservoir to overcome tumor hypoxia	^385^
Gd_2_O_3_-MS NS	Ultrasmall Gd_2_O_3_ NPs (hydrodynamic diameter: 1.5 ± 0.1 nm)-encapulated MSN with an Au shell	MRI imaging and PTT of prostate cancer	Gd_2_O_3_ NPs as the MRI contrast agent, and Au shell as the photothermal agent	^392^
MSNs-ABC@PDA-OVA	ABC-loaded, PDA-coated, and OVA-linked MSNs (particle size: 251.88 ± 1.91 nm)	Treatment of melanoma	Combined PTT and immunotherapy based on photothermal PDA and model antigen OVA	^393^
MMSN/GQDs	Graphene quantum dots-capped and Fe_3_O_4_-loaded MSN (hydrodynamic diameter: 161 ± 19 nm)	Treatment of breast cancer	GQD and Fe_3_O_4_ for PTT and magnetic hyperthermia therapy, respectively	^397^
AuNCs@mSiO_2_@MnO_2_	AuNCs-loaded and MnO_2_ nanosheets-wrapped mSiO_2_	Treatment of breast cancer	MnO_2_ as a catalase mimic to enhance AuNCs-medicated PDT efficiency	^407^
HMONs-MnPpIX-PEG	MnPpIX-loaded PEGylated HMONs	Treatment of breast cancer	MnPpIX as a sonosensitizer for SDT	^412^
Silica-DOX@Chitosan-Pt	Pt NPs-attached and DOX-loaded chitosan-silica hybrid	Treatment of breast cancer	Pt NPs-triggered EDT and DOX-mediated chemotherapy	^141^
OMVs-MSN-5-FU	Gram-negative bacteria OMV-coated and 5-FU loaded MSN (particle size: 140 nm)	Treatment of oral squamous cell carcinoma	OMV for regulating the immune response, and 5-FU for anti-tumor by inhibiting the activity of thymine nucleotide synthase	^516^
TTO@CTAB@MSNs	TTO-encapsulated CTAB micelle as template to prepare MSNs with particle size below 600 nm	In vitro antibacterial therapy	Synergistic antibacterial effect of TTO and CTAB against *E. coli* and *S. aureus*	^370^
MSN-EuGd-PEGMA@Cur	Eu^3+^ and Gd^3+^-doped MSN surface modified with PEGMA and loaded with Cur	Treatment of Zika virus infection	Eu^3+^ and Gd^3+^ for FL and MRI imaging, MSN-protected Cur for treating Zika virus	^371^
MSNs-NH_2_-SH-QR2	Amino groups-modified MSNs loaded with SH and QR to form nanoformulations (particle size: 236 ± 18.8 nm)	Inactivating H5N1 virus	Antiviral prodrug compounds SH and QR achieve strong virucidal effect synergistically, and induce strong anti-inflammatory effect	^372^
PSiO_2_-NGF	NGF-loaded PSiO_2_ films (~20 µm thick)	Treatment of Alzheimer’s disease	Degradable PSiO_2_ delivers neuroprotective NGF for decreasing the cholinergic neurons loss	^374^
MSN-CCM	CCM-loaded MSN (particle size: 115.63 ± 0.90 nm)	Treatment of Alzheimer’s disease	CCM can revert the cognitive deficit in mice	^375^
L-AMSN	Atorvastatin-loaded lipid bilayer-coated MSN	Treatment of acute kidney injury	Atorvastatin decreases the malondialdehyde, pro-inflammatory cytokines, and superoxide dismutase levels	^376^
miR-MSNs	miR-loaded MSNs (particle size: 108.8 ± 0.6 nm)	Treatment of lipid metabolic disorders	MSNs deliver miR to decrease the serum triglyceride level and hepatic steatosis	^377^
N-EDMSNs	FGF-21 plasmids and liraglutide-loaded N-EDMSNs with open large pore (>10 nm) and small mesopores (~2.5 nm)	Treatment of metabolic diseases	N-EDMSNs increase the transfection efficiency, and the pFGF21and liraglutide show synergistic effect in reducing blood glucose and body weight	^378^
MSNs-PA@PEI	PA-loaded and PEI-coated MSNs	Treatment of osteoporosis	Co-delivering siRNA plasmid and osteogenic peptide to stimulate the osteogenic markers and improve the bone microarchitecture	^379^
MS-CeO_2_-miR129	CeO_2_ NPs-immobilized and miR129-loaded MSNs (particle size: 60–100 nm)	Treatment of radiation-induced skin injury	CeO_2_ and miR129 for ROS elimination and anti-inflammation by activating PARP/γh2ax signaling pathway	^431^
nSC scaffold	porcine demineralized cancellous bone-derived porous nSC with inherent hierarchical pore	Bone regeneration	nSC scaffold provides a biomimetic microenvironment to promote host MSC recruitment, proliferation and osteogenesis	^488^
SAL@MSNs/Gelatin/C-PEEUU	SAL-loaded MSNs dispersed in Gelatin, and coated by C-PEEUU	Blood vessel implantation	Promoting cell proliferation, and preventing intimal hyperplasia	^489^
nHAp@MSN	nHAp (particle size: <50 nm)-loaded MSN (particle size: 150–350 nm)	relieving dentin hypersensitivity.	MSNs enhance the acid stabilization of nHAp and allow it to form stable crystal deposits in the demineralized portion of the teeth	^498^

*cDNA* carcinoembryonic antigen, *CEA* carcinoembryonic antigen, *CUR* curcumin, *ZnO* zinc oxide, *pAbs* polyclonal antibodies, *UCNPS* up-converting nanoparticles, *CRP* C- reactive protein, *4-ATP* 4-aminothiophenol, *Apts* aptamer, *MB* methylene blue, *MPSF* mesoporous silica film, *RBD* receptor-binding domain, *FITC* fluorescein isothiocyanate, *HE* hydroethidine, *GSH* glutathione, *CD* carbon dot, *DFNS* dendritic fibrous nano-silica, *SH* alkyl thiol, *VMSN* vanadium-incorporated dendritic mesoporous silica, *SNP* silica nanoparticles, *PET* positron emission tomography, *CT* X-ray computed tomography, *PFH* perfluorohexane, *PDA* polydopamine, *US* ultrasound, *Cy754* NIR dye, *SLNs* sentinel lymph nodes, *MTX* mitoxantrone, *ICG* indocyanine green, *FMSNs* superhydrophobic mesoporous silica nanoparticles, *DOX* Doxorubicin, *PFDTS*, fluoroalkylsilane with a chemical formula of F_3_C(CF_2_)_7_(CH_2_)_2_–Si(OC_2_H_5_)_3_, *D-MON* deformable mesoporous organosilica nanoparticles, *RGD* arginyl–glycyl–aspartic acid, *Gd-DTPA* Magnevist, *PL* photoluminescence, *MSNs-C* cell membrane-coated MSNs, *Au@MMSN* metal manganese-doped Au core MSN, *Ald* alendronate, *AuNR@MS* mesoporous silica-coated Au nanorod, *DLMSN* dendritic large pore MSN, *CuS* copper sulfide, *RBCc* red blood cell content, *RBCm* red blood cell membrane, *PTT* photothermal therapy, *OVA* ovalbumin, *ABC* ammonium bicarbonate, *MMSN/GQDs* graphene quantum dots-capped magnetic MSN, *AuNCs* Au nanoclusters, *PDT* photodynamic therapy, *HMONs* hollow mesoporous organosilica nanoparticles, *MnPpIX* manganese protoporphyrin, *SDT* sonodynamic therapy, *EDT* electrodynamic therapy, *OMV* outer membrane vesicles, *5-FU* 5-Fluorouracil, *TTO* tea tree oil, *CTAB* cetyl trimethyl ammonium bromide, *PEGMA* Poly(ethylene glycol) methacrylate, *Cur* curcumin, *SH* shikimic acid, *QR* quercetin, *PSiO*_*2*_ porous SiO_2_, *NGF* nerve growth factor, *CCM* curcumin, *L-AMSN* Atorvastatin (ATV)-loaded lipid bilayer-coated MSN, *miR* MicroRNA-33, *N-EDMSNs* amino-functionalized and embedded dual-MSNs, *FGF-21* fibroblast growth factor 21, *PA@PEI* targeting ligand@poly(ethylenimine), *nSC* nanosilica-collagen, *MSC* mesenchymal stem cell, *SAL* salvianic acid, *C-PEEUU* poly(ester-urethane)urea, *nHAp* nano-hydroxyapatite

### Biosensing

Biosensing technology provides a simple, convenient and fast method for basic medical research and clinical diagnosis. In MSNs-based biosensing applications, the functionalized MSNs matrix and the receptor or indicator embedded in the MSNs together consist of a biosensor.^342^ The principle of sensing is to detect the changes in optical or electrical signal of some specific analytes. Of these, the optical signal is easier to detect and more sensitive. The detection of different kinds of biological targets (glucose, glutathione, amino acids, proteins, bacteria, viruses, etc.) can be achieved by various optical detection means,^343–348^ such as naked-eye colorimetric detection, UV-Vis spectroscopy, fluorescence spectroscopy and Raman spectroscopy based on the MSNs-based nanosensors. MSNs have multiple roles in biosensing applications. On the one hand, MSNs matrix has the capability to enhance the physiological stability of the receptor or indicator, resulting in increased sensitivity and detection rate. On the other hand, as the location where the reaction takes place, MSNs provide some reaction chambers or facilitate interfacial interactions through ordered mesoporous structures.^342^ Researches on MSNs-based nanosensors have been ongoing for the past two decades. In a recent study, researchers achieved the bacterial quantitative determination via aptamer-gated aminated MSNs.^346^ During the construction of biosensor, the 4-aminothiophenol (4-ATP) signal molecules were firstly encapsulated into the pores of MSNs, and then the negatively charged aptamers were connected to the pore entrance. After adding the *Staphylococcus aureus*, the aptamers gatekeepers were specially separated and 4-ATP molecules were released, which could be detected by Raman spectroscopy for analyzing the concentration of *Staphylococcus aureus*.^346^ In another study on aptamer-gated MSNs nanosensors, Tabrizi et al. encapsulated the methylene blue into the pores of MSNs, and developed an electrochemical biosensor for detecting the receptor-binding domain of SARS-CoV-2. The electrochemical signal increased with decreasing methylene blue concentration, and the biosensor thus constructed exhibited good stability, sensitivity and selectivity, providing a new tool for the early detection of SARS-CoV-2.^349^

### Bioimaging

Bioimaging is a powerful tool that can be used for the diagnosis of various diseases, and the NPs-based contrast agents have become the research frontier in the field of bioimaging.^350–352^ The integration of contrast agents onto MSNs can address the problems of insufficient stability and poor water solubility of contrast agents, as well as the targeting ligand modification on the surface of MSNs can enhance the enrichment of contrast agents at selected sites.^353,354^ In magnetic resonance imaging (MRI), the mesoporous structure of MSNs allows the free access of water molecules in the MSNs matrix, thus enhancing the contrast performance of Fe/Mn/Gd-involved MSNs-based nanoprobes.^353^ In fluorescence imaging (FL), the protective effect of MSNs can prevent the dye self-aggregation or self-quenching, improves the photobleaching resistance property and enhances quantum yields.^354^ Positron emission tomography (PET) imaging is a noninvasive imaging modality and often applied in disease diagnosis. The molecular probes used in PET imaging need to be labeled with the radioisotopes, e.g., ^64^Cu and ^18^F. The introduction of MSNs matrix offers great potential in extending the half-life of radioisotopes.^355–357^ In photoacoustic (PA) imaging, the PA contrast agents such as polydopamine (PDA) NPs,^358^ Cy754,^359^ and ICG^360^ have also been demonstrated to be protected by MSNs matrix, thus to obtain desirable PA signals. In addition to those listed above, MSNs have been widely applied in ultrasound imaging,^361,362^ X-ray computed tomography (CT) imaging,^168,363^ or multimodality imaging.^364–366^

### Targeted disease therapy

**Fig. 7:**
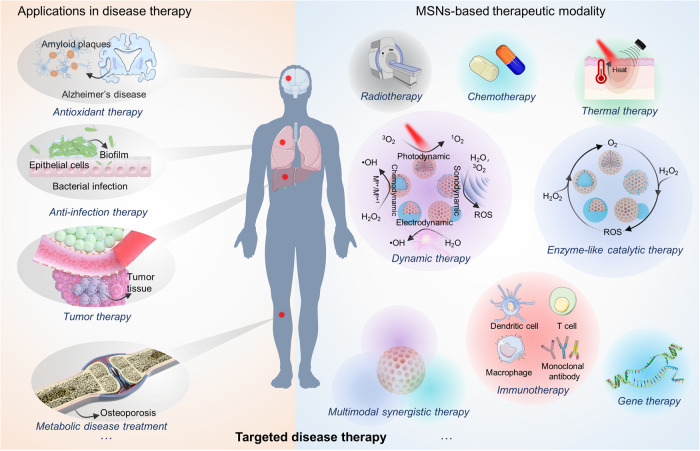
Schematic illustration of MSNs in targeted disease therapy. MSNs-based nanoplatforms are utilized in antioxidant therapy (e.g., Alzheimer’s disease), anti-infection therapy (e.g., bacterial infection), tumor therapy, or metabolic disease treatment (e.g., osteoporosis). The current developed MSNs-based therapeutic modalities include radiotherapy, chemotherapy, thermal therapy, dynamic therapy, enzyme-like catalytic therapy, immunotherapy, gene therapy, and multimodal synergistic therapy. The image elements were created using Autodesk 3ds Max and Servier Medical Art (https://smart.servier.com)

The unique properties of MSNs make it highly superior for the construction of multifunctional nanocomposites and drug delivery system. Different therapeutic nanoplatforms obtained through the careful design of MSNs have shown promising potential in the field of targeted disease therapy. Current applications of MSNs include tumor therapy,^367–369^ anti-infection therapy (bacteria and virus),^370–373^ anti-oxidant/anti-inflammatory therapy (Alzheimer’s disease, acute kidney injury, etc.),^374–376^ and metabolic diseases treatment (diabetes, osteoporosis, fatty liver, etc)^377–380^ (Fig. 7). In general, the targeted therapeutic characteristics of MSNs can be reflected in the following aspects: (1) Loading chemotherapy drugs or other drugs with toxic side effects, so that they can be released in a controlled manner by MSNs at the targeted sites to prevent their premature release. (2) Selective enrichment at the lesion sites through passive targeting or active targeting, resulting in the higher therapeutic efficacy. (3) Use the MSNs matrix to develop a non-invasive, spatial and temporal controllable targeted therapeutic strategy, thus to overcome the limitations and defects of conventional clinical treatment modalities. To date, the emerging targeted therapeutic modalities developed based on MSNs include dynamic therapy such as photodynamic, sonodynamic and chemodynamic therapies, thermal ablation therapy such as photothermal therapy and magnetic thermotherapy, enzyme-like catalytic therapy, immunotherapy, gene therapy, and others. Meanwhile, the integration of various therapeutic modalities on a single MSNs is expected to enhance the therapeutic efficacy synergistically (Fig. 7). In this part, we will discuss MSNs-based therapeutic modalities.

#### Chemotherapy and radiotherapy

Chemotherapy and radiotherapy are routine clinical treatments. In chemotherapy, numerous chemotherapeutic agents such as doxorubicin, camptothecin and cisplatin have proven to be very effective in the treatment of malignant tumors, but they suffer from low solubility in the aqueous systems, poor physiological stability, and hard intravenous administration. To overcome these obstacles, researchers have been making a variety of attempts. Back in 2007, Lu et al. tried to encapsulate the anti-tumor drug camptothecin into the pores of fluorescent MSNs, and the resulting camptothecin-loaded MSNs showed remarkably growth inhibition of human cancer cells including pancreatic cancer cells, colon cancer cells and stomach cancer cells, compared with free camptothecin.^381^ And since chemotherapy has been associated with high side effects and poor patient compliance, more research has focused on combining chemotherapy with other treatment modalities, e.g., chemo-photothermal therapy based on Pd/MSNs nanocomposite,^137^ synergistic electrodynamic-chemotherapy based on Pt/MSNs nanocomposite,^141^ chemodynamic-chemotherapy based on Mn/Au/MSNs nanocomposite.^382^ In radiotherapy, Au NPs have been widely studied as a radiosensitizer because of their high X-ray absorption coefficient, and MSNs are often used to improve the stability, non-specific interactions and toxicity of Au NPs.^383,384^ A study reported by Chen et al. demonstrated that the gold-nanorod-seeded MSNs could avoid the undesired aggregation in the physiological medium, and act as a highly efficient radiosensitizer for the radiotherapy of oral squamous carcinoma with high therapeutic index.^384^ Of course, the combination of radiotherapy with other treatment modalities has been also extensively studied to offer higher therapeutic outcome. For example, cell membrane-coated, and CuS-loaded MSNs were designed by Wu et al. for synergistic photothermal-radiotherapy,^385^ selenadiazole derivative-loaded and folic acid-modified MSNs reported by Liu et al. for synergistic cervical cancer chemo-radiotherapy.^386^

#### Thermal therapy

The common thermal therapy strategies include PTT and magnetothermal therapy (MTT). The integration of some certain functional components into the MSNs can induce photothermal or magnetothermal effects by external light irradiation or by applying a magnetic field, respectively. During the thermal therapy, the locally generated thermal effect can cause irreversible damage to cells or tissues at the lesion, thus achieving targeted, non-invasive treatment. In terms of PTT, organic photothermal agents (PTAs) such as phthalocyanines and porphyrins can convert light energy into heat energy through non-radiative forms of decay-vibrational relaxation,^387,388^ as well as inorganic PTAs such as noble metals achieve photothermal conversion through LSPR effect,^389,390^ to produce the temperature required for treatment. The single MSNs-based PTT modality has been thoroughly studied, and the current research focuses on the construction of integrated nanoplatform for diagnosis and treatment, or the development of multimodal synergistic treatment strategies.^391^ A recent study reported by Kadria-Vili et al. demonstrated that MSNs could encapsulate Gd_2_O_3_ NPs for dual T_1_/T_2_ contrast and Au nanoshell PTA for NIR light-responsive PTT, thus giving clinicians the ability to “see and treat”.^392^ In addition, MSNs were considered as a biocompatible and multifunctional nanoplatform for photothermal-immunotherapy against melanoma tumors, through simply integrating polydopamine PTA, ovalbumin model antigen, and ammonium bicarbonate antigen release promoter.^393^ PTT suffers from insufficient penetration depth of NIR light, resulting in decreased treatment efficiency. In contrast, MTT is able to kill deep tumor cells by increasing the temperature to 43–48 °C, without causing significant side effects to surrounding normal cells.^394^ During the action, the magnetic NPs act as thermoseeds, exposed to an alternating magnetic field, absorbing magnetic energy and dissipating thermal energy through the magnetic relaxation effect.^395,396^ In a study reported by Yao et al., MSNs-based nanoplatform was proven to achieve synergistic therapeutic effect of PTT and MTT. They utilized MSNs shell to coat magnetic Fe_3_O_4_, and served graphene quantum dots with photothermal conversion capacity as the cap to control DOX release. After reaching the tumor acidic microenvironment and applying external light and magnetic field stimuli, DOX was released in a controlled manner and the accelerated tumor hyperthermia was achieved.^397^ In addition to PTT and MTT, the MSNs-mediated radiofrequency thermotherapy is also reported. Especially, the Si NPs themselves can serve as an excellent sensitizer to enhance the radiofrequency radiation effect.^398^ A study by Tamarov et al. demonstrated that the radiofrequency radiation could more efficiently trigger temperature-responsive drug release than infrared light, thus inhibiting tumor cell growth even after one treatment.^399^

#### Dynamic therapy

Dynamic therapy refers to an emerging therapeutic modality in which nanosensitizers are activated in the presence of exogenous stimuli such as light, ultrasound and electric field or endogenous small molecules such as H_2_O_2_, and sequentially induce the in situ production of toxic free radicals for damaging important cellular components (e.g., lipids, proteins or DNA), thus leading to cell apoptosis or necrosis.^400,401^ Typical dynamic therapy strategies include photosensitizer-mediated PDT, sonosensitizer-mediated SDT, nanocatalyst-mediated CDT and electrodynamic therapy (EDT).^401^ In dynamic therapy processes, MSNs are excellent substrate materials for loading nanosensitizers, and their roles include enhancing nanosensitizer stability, reducing potential toxicity, providing a suitable microenvironment for chemical reactions, and acting as a therapeutic nanoplatform to integrate multifunctional components. For example, PDT is an oxygen-dependent therapeutic modality, and its therapeutic efficiency is affected by the oxygen concentration. However, the tumor microenvironment or biofilm microenvironment are always characterized with hypoxia.^402–404^ For this reason, the simultaneous integration of some components with oxygen-producing functions, such as catalase,^405,406^ MnO_2_,^407,408^ and Pt NPs,^409,410^ into the pores or surfaces of MSNs is expected to enhance the photodynamic performance. Similar to PDT, SDT is also a non-invasive therapeutic modality and many of sonosensitizers are originated from photosensitizers,^411^ such as Mn protoporphyrin-encapsulated biodegradable MSNs for MRI-guided tumor SDT,^412^ and IR-780 sonosensitizer-loaded hollow MSNs for SDT of pancreatic cancer.^413^ Differently, a very unique feature in MSNs-based SDT is that MSNs themselves are potentially effective sonosensitizers with the ability to enhance ultrasound-induced cavitation effect, without the introduction of additional sonosensitizers.^414^ The main reason for this is that MSNs possess large amounts of hydrophobic mesopores that can serve as bubble nucleation seeds in response to low-intensity ultrasound.^415,416^ CDT exploits the disease microenvironment to activate the Fenton/Fenton-like reaction to produce strongly oxidizing •OH for specific and targeted disease therapy, which is first proposed by Zhang et al. in 2016,^417^ and has subsequently attracted much attention from researchers.^418,419^ The efficiency of CDT is closely related to the H_2_O_2_ content in the disease microenvironment, so researches have focused on introducing functional components on MSNs matrix to enhance the content of H_2_O_2_ reactants, thus increasing the yield of •OH. In this regard, Li et al. encapsulated both ultra-small CaO_2_ and Fe_3_O_4_ NPs in dendritic MSNs. In the acidic tumor microenvironment, CaO_2_ reacts with H^+^ to produce large amounts of H_2_O_2_, leading to the enhancement of Fe_3_O_4_-mediated Fenton reaction.^420^ In another study, natural glucose oxidase was used to consume intratumoral glucose while generating additional H_2_O_2_ for the subsequent Fenton reaction catalyzed by Fe_3_O_4_ NPs.^421^ Besides, EDT is an emerging therapeutic approach newly proposed in 2019.^422^ In this pioneering work, Pt NPs, assisted by a square-wave alternating current electric field, induced the breakdown of water molecules on their surface to generate cytotoxic •OH, which effectively inhibited tumor cell proliferation and triggered tumor cell apoptosis.^422^ Regarding the MSNs-based EDT, Gu et al. designed a Pt/MSNs nanocomplex for the first time and encapsulated DOX into MSNs pore channels, followed by a layer of chitosan to prevent the premature drug release. Under the tumor microenvironment and alternating current electric field, the designed MSNs-based nanoplatform effectively eliminated large-sized tumors (over 500 mm^3^) while ensuring minimal side effects. This work is the first study to combine EDT and chemotherapy, and it provided new insights into the development of MSNs-based EDT nanoplatforms.^141^

#### Enzyme-like catalytic therapy

In this process, nanozymes are capable of following enzyme kinetics under physiological conditions and catalyzing the conversion of enzyme substrates by mimicking the structure or function of natural enzymes. Current nanozymes are dominated by oxidoreductase activity, i.e., oxidative stress amplification-related POD or OXD mimics,^423,424^ and antioxidation-related CAT or SOD mimics.^425,426^ By designing nanozymes with various catalytic activities, the redox balance of cells can be precisely regulated, i.e., boosted ROS generation or ROS scavenging, enabling symptomatic treatment in a variety of disease models.^427–430^ Due to the rich and tunable pore structure, MSNs have become an excellent nanoreactor in enzyme-like catalytic therapy.^431–435^ In a study by Wu et al., MSNs were developed as a compartmental hierarchical nanoreactor for multi-pathway generation of ^1^O_2_. Specifically, they designed a compartmental multienzyme nanoreactor with penetrated super cavity and connected dual mesoporous channels for the encapsulation of a multi-enzyme complex (SOD-lactoperoxidase (LPO)) and a photosensitizer ICG molecule. The cascade biocatalysis and enzyme-enhanced photosensitization could occur in parallel, due to the unimpeded substrate diffusion between SOD and LPO and reduced external diffusion effects. This strategy offers unique advantages in the treatment of hypoxia tumors.^436^ In terms of MSNs-based ROS elimination platform, Purikova et al. filled ultrasmall cerium dioxide (CeO_2_) NPs with superior colloidal stability into the pore channels of MSNs, and functionalized the MSNs surface with ROS-responsive methylthiopropyl groups. The released CeO_2_ NPs could scavenge more than 80% of H_2_O_2_ within 10 min in an ROS-excess environment, which was demonstrated to be a potentially safe and efficient antioxidant therapeutic agent.^437^

#### Immunotherapy

Immunotherapy is a therapeutic approach implemented using the immune system of biological organisms, and can be carried out by activating or suppressing the immune system.^438,439^ The most common application is cancer immunotherapy.^440,441^ In cancer therapy, immunotherapeutic agents such as tumor-associated antigens or immune adjuvants are often used to educate antigen-presenting cells and T cells, thus to enhance the host immune response to cancer. However, it remains a challenge to improve the delivery efficiency of immunotherapeutic agents, to avoid dose-dependent toxicity, and to mitigate immune-mediated adverse effects.^438,439^ In this regard, MSNs have emerged as an ideal multifunctional platform for improving immunotherapy because of their excellent porosity, good biocompatibility and ease of surface modification.^30,31^ In MSNs-based immunotherapy, MSNs can play a role in different periods of the cancer immune cycle.^31^ For example, chemokine-loaded MSNs promote T-cell tumor chemotaxis,^442^ MSNs-based platforms regulate the immune checkpoint proteins,^443^ MSNs loaded with chemotherapeutic drugs,^444^ photosensitizers,^445^ sonosensitizers^446^ or PTAs^447^ induce immunogenic cell death (ICD), and MSNs as vaccine vehicles are utilized to deliver antigens and adjuvants. In particular, MSNs themselves can serve as effective adjuvants, and contribute to the recruitment and activation of immune cells.^448–450^ At present, there has been considerable progress in MSNs-based immunotherapy platforms. A few recent studies are presented as brief examples. Large-pore MSNs-coated upconversion NPs are used as immune adjuvants to deliver photosensitizers merocyanine 540, model proteins, and tumor antigens for synergistic photodynamic immunotherapy of cancer.^122^ The diselenide-bridged organic MSNs loaded with chemotherapeutic ruthenium compound were acted as potential ICD nanoamplifiers for improved cancer chemo-immunotherapy.^451^ Au, BP co-loaded MSNs modified with macrophage cell membrane were applied to deliver CO precursor for improving SDT-induced ICD effect and inhibiting the growth and metastasis of breast tumor.^446^

#### Gene therapy

Gene therapy refers to the introduction of exogenous genes into the target cells to treat diseases by replacing defective genes or adding new genes.^452,453^ In many cases, the core of gene therapy lies in the selection of a suitable gene vector to efficiently deliver the gene to the target cells.^454,455^ Viral vectors are frequently used in clinical settings, and although they have high efficiency in gene transfection, their safety issue, i.e., causing adverse immune response in the body, cannot be ignored.^454^ MSNs, as excellent multifunctional non-viral vectors, have advantages in biocompatibility, tissue toxicity, and targeting.^456,457^ As early as 2004, MCM-41-type MSNs were used as gene transfection reagents for delivery of plasmid DNA.^52^ Since then, many different gene therapy strategies have been developed based on MSNs, and their therapeutic effects have been validated in the fields of tumor therapy,^458–460^ diabetes treatment,^378^ and wound healing.^461,462^ As an example, suicide gene therapy as a gene-mediated enzyme prodrug treatment, is able to kill tumor cells specifically after in situ conversion of the drug into toxic drugs.^463^ To control the synergistic intracellular release of suicide genes and prodrugs and enhance therapeutic efficacy, Wang et al. investigated the efficacy of spherical and rod-shaped magnetic MSNs in targeted drug delivery, and gene transfection, MRI imaging, and hepatocellular carcinoma treatment. It was demonstrated that the Janus-type rod-like magnetic MSNs loaded with ganciclovir (GCV) and functionalized by PEG-g-PLL showed better drug-loading capability and faster drug release behavior, indicating that they are a useful tool to construct the herpes simplex virus thymidine kinase/ganciclovir (HSV-TK/GCV) gene therapy system.^464^

#### Multimodal synergistic therapy

The complexity and heterogeneity of the disease microenvironment leads to the problem of poor therapeutic efficiency of single therapies.^465–469^ The current clinical research trend has gradually shifted to multimodal synergistic therapy, as we have mentioned above with many examples of multimodal synergistic therapy platform construction. In multimodal synergistic therapy, the interaction between different treatment modalities can significantly improve the shortcomings of single therapy, such as overcoming the lack of light penetration depth in PDT, reducing the toxic side effects of chemotherapy, etc., and generating stronger therapeutic outcome through superimposed effects (namely “1 + 1 > 2”).^470–472^ A prominent feature of multimodal synergistic therapies is the ability to effectively overcome multidrug resistance in diseases, and MSNs are uniquely advantageous in integrating these aforementioned treatment modalities.^369,473–475^ As an example, most of the multidrug resistance generated during chemotherapy of malignant tumors might stem from the overexpression of transmembrane ATP-binding cassette transporter and transmembrane Ca^2+^ channels.^476,477^ To address this problem, researchers used mesoporous silica nanocapsules to encapsulate and deliver the cytotoxic drug DOX and T-type Ca^2+^ channel siRNA.^478^ On the one hand, MSNs can bypass the drug efflux pumps and enter cells directly through endocytosis, increasing the intracellular accumulation of drugs. On the other hand, siRNA can knock down the T-type Ca^2+^ channel, leading to a decrease in cytosol Ca^2+^ concentration in the cytosol, thus increasing the sensitivity of tumor cells to DOX. Accordingly, the as-designed MSNs-based therapeutic system achieved effective chemotherapy-gene therapy to overcome the multidrug resistance in breast cancer.^478^ Besides, drug-resistant bacterial infections are a current thorny public health issue.^479^ In this regard, researchers have developed a photodynamic combined lysozyme antimicrobial therapy based on upconversion NPs/MSNs nanocomposites to combat the problem of drug-resistant bacterial infections in deep tissues. Briefly, upconversion NPs were sequentially encapsulated with hierarchical coating of dense silica and dendritic mesoporous silica, followed by loading photosensitizers and lysozyme, and finally modified with bacterial hyaluronidase-response valves on the MSNs surface to achieve controllable release of lysozyme. The results showed that this system promoted direct attack of ROS on cell membranes and cytoplasm through enzymatic cell wall disassembly, resulting in an excellent bactericidal rate toward methicillin-resistant *Staphylococcus aureus* (> 5 log10 viability reduction).^480^

### Tissue engineering

In addition to being widely used in targeted disease therapy, MSNs are emerging in the field of tissue engineering.^341,481^ Tissue engineering refers to the fabrication of bioactive scaffolds by technical means for restoring, maintaining and enhancing the function of damaged tissues and organs.^481^ The ideal bioactive scaffolds should have excellent biocompatibility and good interaction with cells. The unique advantages of MSNs can be well combined with scaffolds to enhance the bioactivity by utilizing the carrier nature of MSNs to achieve controlled release of bioactive agents. In particular, the inherent bioactivity of Si ions gives them an advantage in repair-related regenerative medicine.^341,482^ Up to now, MSNs have made great research progress in many aspects of tissue engineering, especially in bone tissue engineering.

MSNs-based scaffolds are able to enhance the cell proliferation, migration, adhesion and differentiation in bone tissue engineering. Many studies have systematically investigated and revealed the role of MSNs-based scaffolds in the regulation of cellular functions.^483–486^ Bone defects-involved regenerative repair is difficult to obtain a desired outcome due to the delay of early vascularization and poor osteogenic activity of bone implants. In this regard, researchers have designed a polycaprolactone network containing vascular-like structure, and introduced a nanofibrous gelatin-silica scaffold into it. Dimethyloxalylglycine (DMOG) and peptide-1 (BFP) were then loaded into MSNs, and the differential spatial distribution and sequential release of DMOG and BFP allowed the bioactive scaffold to promote angiogenesis by stimulating the migration of human umbilical vein endothelial cells (HUVECs), the tube formation and the expression of angiogenesis-related genes/proteins (HIF-1α, e-NOS, KDR and VEGF), as well as enhance osteogenesis by upregulating the expression of bone marrow-derived mesenchymal stem cells (BMSCs)-related osteogenic genes (Runx2, Col I, OPN and OCN) and promoting mineral matrix formation.^487^ Besides, Mora-Raimundo et al. reported MSNs as a nanocarrier to deliver SOST small interfering RNA (siRNA) and osteostatin, and the as-designed system could be used for osteoporosis remission via improving the bone microarchitecture.^379^ Wang et al. designed a biomimetic silica-collagen scaffold via a robust biosilicification strategy, and the silica-collagen scaffold could mimic the unique microenvironment of bone extracellular matrix (ECM), resulting in the improvement of mesenchymal stem cell (MSC) recruitment and bone repair.^488^ These MSNs-based scaffolds have great potential for clinical translation in the treatment of bone defects.

A few studies on MSNs as a nanocarrier to deliver drugs for vascular tissue engineering were also reported. To promote the early angiogenesis in revascularization, Guo et al. developed salvianic acid-loaded MSNs and subsequently doped into a bilayered gelatin/polyurethane tubular scaffold. The addition of salvianic acid-loaded MSNs allowed the tubular scaffold to show better cell proliferation capacity and anticoagulant function.^489^ In another study, researchers constructed a bilayered vascular graft, which consisted of the inner layer Poly(lactic-co-glycolic acid) (PLGA)/Collagen (PC) nanofibers and the outer layer polyurethane (PU) nanofibers. The heparin-loaded MSNs were modified into the inner layer for promoting cell proliferation and blood compatibility. The hematoxylin-eosin and immunohistochemical staining experiments indicated the regeneration of monolayer endothelium and smooth muscle on the vascular grafts, demonstrating their feasibility in serving as useful blood vessels with long-term patency.^490^ In addition, in wound healing, MSNs contribute to promoting healing efficiency through similar effects as discussed above. One difference, however, is that MSNs can also deliver some antioxidant components for ROS scavenging,^491,492^ since the over-expression of ROS at the wound site can slow down the healing process.^493,494^

In oral tissue engineering, MSNs can act as a noninvasive vehicle for oral rehabilitation.^495^ Typically, oral disease such as dental caries and dentin hypersensitivity can be well treated by constructing stimu-responsive delivery nanoplatforms. Chlorhexidine (CHX) is a broad-spectrum antimicrobial agent, and often used in oral rehabilitation. To enhance its antibacterial effect, CHX-loaded MSNs are modified into dentin adhesives. Under the acidic environment caused by cariogenic bacteria, CHX can be released by pH responsiveness, thus inhibiting the formation of cariogenic biofilm.^496,497^ To achieve effective treatment of dentin hypersensitivity, MSNs-based biomaterials are used to block the dentinal tubules to reduce their sensitivity to chemical and physical stimuli. For example, in nano-hydroxyapatite@MSNs complexes, MSNs enhance the acid stabilization of nano-hydroxyapatite and allow it to form stable crystal deposits in the demineralized portion of the teeth.^498^ Ag-based bioactive glass nanoparticles@MSNs are used to treat dentin hypersensitivity by blocking dentinal tubules and promoting soft tissue regeneration.^499^

## Clinical translation

Clinical trials involving mesoporous silica have been ongoing since 2007, exploring its potential in various biomedical applications (Table 3). To date, the clinical trials have demonstrated the safety and good tolerability of silica in human subjects. In 2014, MSNs as oral delivery carriers for enhancing the pharmacokinetic profile of drugs with limited aqueous solubility were reported.^500^ A silica NPs-lipid hybrid formulation loading with ibuprofen (Lipoceramic-IBU) was prepared. The randomized, double-blind, single-dose oral administration study (20 mg ibuprofen) was conducted on 16 healthy male volunteers. The bioavailability of Lipoceramic-IBU was found to be 1.95 times higher than that of the commercial tablet Nurofen®.^500^ This result suggested the safe and effective use of silica-based NPs in mimicking food effects to optimize the oral absorption of drugs with poor water solubility. In another clinical trial consisting of 12 participants in 2016, the bioavailability of ordered MSNs-based fenofibrate formulation increased by 54.1% after a single oral dose, when compared to the commercial formulation Lipanthyl®.^39^ Due to the potentially ideal clinical outcomes of silica-based nanocarriers, the pharmacokinetics, safety, and metabolic profile of a hybrid core-shell silica NP (named Cornell dots, C dots) with a diameter of 6 nm functionalized with ^124^I, targeting peptide cyclo-(Arg-Gly-Asp-Tyr), and Cy5 (^124^I-cRGDY–PEG–C’ dots) is first conducted in humans using PET imaging and clinical tracer at clinical trials in 2014 (NCT01266096).^58^ Results showed the good safety and reproducible PK signatures of C dots with the whole-body clearance half-time of 13–21 h after intravenous administration in patients, which is shorter than ^111^In labeled liposomes and ^131^I-labeled humanized monoclonal antibody A33. These results verified the possibility of these NPs for cancer diagnostics in humans.^58^ Similarly, 8 nm core-shell silica NPs encapsulated with Cy5.5 in the core and connected with cRGDY on the surface (cRGDY-PEG-Cy5.5-nanoparticles) were used in patients at nanomole doses for sentinel lymph nodes detection of head and neck melanoma at a phase 1/2 human clinical trial.^501^ The remarkable safety, accuracy, reliability, and high contrast of these particles for visual identification of sentinel lymph nodes are irreplaceable by other available imaging molecules currently, of which phase 2 clinical trial is ongoing and will be finished in 2024 (NCT02106598). In the meantime, another two clinical trials based on C dots are carried out. In 2018, the ^89^Zr-DFO-cRGDY-PEG-Cy5-C’ dots tracers were first used in both surgical and non-surgical patients for PET-CT imaging of malignant brain tumors in phase 1 clinical trial (NCT03465618). The distribution and removal profiles from the body of these particles will also be assessed till 2023. In 2019, ^64^Cu-NOTA-PSMA-PEG-Cy5.5-C’ dots tracer was used in another human phase 1 clinical trial for guiding the surgery of prostate cancer by PET and MRI imaging (NCT04167969).As a nanocomposite with high safety and excellent photothermal conversion efficiency, the silica-gold NPs have also been demonstrated to be potential therapeutic agents for plasmonic photothermal therapy of atherosclerosis (NCT01270139),^502,503^ and photothermal ablation of tumors (NCT00848042, NCT04240639).^40^ In addition, an engineered mesoporous silica (SiPore15™) with particle size of 1.1–1.5 μm × 0.2–0.4 μm was developed by Baek et al., and they demonstrated that such pure synthetic amorphous silica could remarkably decrease long-term blood glucose levels, showing great promise for the treatment of prediabetes (NCT03823027).^504^ In other clinical trials, an amorphous silica-containing bioactive scaffold, called Siloss®, was used to promote bone regeneration, and could be replaced by natural bone due to its fully resorbable nature (NCT02639572), and the silica-calcium phosphate nanocomposite (SCPC) was developed as graft material for the reconstruction of the volume-deficient alveolar ridges (NCT05317039). In all, these clinical trials demonstrate the safety and efficacy of MSNs in various biomedical application scenarios.

**Table 3:** Silica-based nanoformulations in clinical trials

Formulation	Silica description	Investigated application/indication	Start year	Clinical trial phase	Status	Identifier
NANOM-FIM	Core/shell silica-gold NPs with size of 60/15–70/40 nm; Fe_3_O_4_ magnetic silica-gold NP with size of 90–150 nm	Plasmonic photothermal therapy of atherosclerosis	2007	Not applicable	Completed	NCT01270139
SiPore15™	Rod-shaped mesoporous silica with particle size of 1.1–1.5 μm × 0.2–0.4 μm	Treatment of prediabetes	2019	Not applicable	Completed	NCT03823027
Porous silica	Rod-shaped silica with particle size of 1–3 μm × 0.4–0.5 μm and pore size in the range of 7–10 nm	Safety issues (effect on gastrointestinal function, bowel emptying habits, and biomarkers)	2015	Not applicable	Completed	NCT03667430
SCPC	Silica-calcium phosphate nanocomposite	Treatment of alveolar bone loss	2021	Not applicable	Active, not recruiting	NCT05317039
Siloss®	Amorphous silica-containing inorganic bone graft material	Treatment of intrabony defects	2014	II	Completed	NCT02639572
^89^Zr-DFO-cRGDY-PEG-Cy5-C’ dots	Dye labeled silica NPs with ultrasmall size	PET imaging of malignant brain tumor	2018	I	Active, not recruiting	NCT03465618
cRGDY-PEG-Cy5.5-C’ dots	Dye labeled silica NPs with ultrasmall size	Fluorescence imaging of head and neck melanoma	2014	II	Recruiting	NCT02106598
^64^Cu-NOTA-PSMAi-PEG-Cy5.5-C’ dots	Dye labeled silica NPs with ultrasmall size	PET/MRI imaging-guided surgical treatment of prostate cancer	2021	I	Recruiting	NCT04167969
^124^I-cRGDY-PEG-C’ dots	Dye labeled silica NPs with ultrasmall size	PET imaging of malignant brain tumor	2011	Not applicable	Active, not recruiting	NCT01266096
AuroShells	Core/shell silica-gold NPs with size of ~150 nm	Photothermal ablation of head and neck cancer	2008	Not applicable	Completed	NCT00848042
		MRI/US imaging-guided photothermal ablation of prostate cancer	2020	Not applicable	Active, not recruiting	NCT04240639

## Conclusion and perspective

With the continuous research and development of nanotechnology in the biomedical fields, the global healthcare nanotechnology market is expected to grow gradually from US$ 17,245 million in 2019 to US$ 252,400 million by 2024.^505^ As an important part of the nanomaterials library, MSNs have become indispensable in the biomedical field due to their easily tunable structure and composition, relatively excellent biocompatibility, and flexible surface functionalization properties. Their footprints have covered various aspects, serving as carriers to deliver various therapeutic agents, as matrixes to construct nanocomposites for meeting specific requirements, or as efficient agents with intrinsic therapeutic effect or bioactivity.

This review provides an overview of the development of MSNs in the biomedical field, highlighting some key research advances, briefly summarizing the types of MSNs developed by different research groups (M41S-series, SBA-series, FDU-series, KIT-series, etc.). In addition, in view of the high versatility of MSNs-based nanocomposites for diagnostic and therapeutic applications, the current MSNs-based architectures and the types of nanocomposites by active elements are summarized. Subsequently, the purpose of surface functionalization of MSNs is discussed. Further, we comprehensively review the biomedical applications of MSNs, including biosensing, bioimaging, targeted disease therapy, and tissue engineering, etc. In particular, targeted therapeutic modalities based on various strategies are meticulously discussed. Finally, the current status of MSNs in clinical trials is presented. It is believed that this review will provide a thorough understanding of MSNs involved in the development history, design, functionalization, and biomedical applications. Despite numerous breakthroughs, the current challenges in MSNs research include three main aspects: systematic toxicity study, construction of simple but efficient MSNs-based nanoplatforms, and in-depth insight into the in vivo action mechanism.

Firstly, although several silica-based nanoformulations have already proceeded to the clinical trial stage and have been shown to be remarkably efficacious and acceptably safe, this is not nearly enough given the tremendous cost researchers have put in upfront. The most important consideration driving the successful clinical translation is a comprehensive and systematic toxicity study of MSNs. Despite the fact that a relatively complete set of toxicity studies on MSNs has been established in the scientific community, a sustained effort is still needed. It is noteworthy that many of the currently designed therapeutic systems for MSNs are disconnected from systematic toxicological evaluations. Some researchers have focused more on the therapeutic effects, but the feasible MSNs-based nanocomposites constructed on this basis are far from the silica NPs used by another part of the researchers in MSNs toxicology evaluation. In future research, under the premise of guaranteeing the efficacy of MSNs-based nanomedicines, more emphasis on the toxicological evaluation and long-term biosafety assessment of MSNs will be more beneficial to promote the possibility of their clinical translation.

Secondly, it is needed to construct simple but efficient MSNs-based nanoplatforms. The abundant characteristics of MSNs have led to their extensive exploitation as multifunctional nanocarriers, and this may be accompanied by the introduction of two or more components into MSNs. Although these multifunctional nanoplatforms have proven to be very effective in numerous papers, the complexity of these platforms does not seem to be friendly to the scale-up preparation and clinical translation. On the one hand, it is well known that the laboratory-level synthesis methods of MSN are very different from those used for industrial scale-up production required for clinical screening and use. The reproducibility and batch stability of nanoformulations are always an important factor to consider in industrial scale-up production, and in this process, simplifying the production steps is the key to promote the industrial scale-up production. However, the complex MSNs-based nanoplatforms require higher nanoformulation preparation technologies and more stringent storage methods, which often require more time, effort, and economic investments. On the other hand, the multiple components may introduce more unsafe factors, it appears to be a huge challenge at this stage to systematically reveal the toxicity mechanisms of these different components when we still do not fully understand the toxicity mechanisms of MSNs. Therefore, it is advocated to slim down the MSNs-based platform as much as possible while meeting the clinical requirements.

Thirdly, an in-depth insight into the in vivo action mechanism of MSNs is needed. The lesion microenvironment has many features that are quite different from normal tissue, and the clinical manifestations of many diseases show heterogeneity in pathology. There is a discrepancy between studies on the action mechanism of MSNs-based nanomedicines at the cellular level and the nanomedicines behavior in the real in vivo environment. In this regard, we need to closely combine different disciplines, research models and characterization tools to deeply reveal the molecular mechanisms of MSNs-based nanomedicines and elucidate the action behavior between materials and biological interfaces.

With the increasing research on MSNs and the continuous efforts to address the aforementioned key scientific problems of MSNs in the biomedical field, it is reasonable to believe that more MSNs-based nanoformulations will move toward clinical trials and achieve successful clinical translation in the future.
